# Chronic Environmental Perturbation Influences Microbial Community Assembly Patterns

**DOI:** 10.1021/acs.est.1c05106

**Published:** 2022-02-01

**Authors:** Lloyd D. Potts, Alex Douglas, Luis J. Perez Calderon, James A. Anderson, Ursula Witte, James I. Prosser, Cécile Gubry-Rangin

**Affiliations:** † School of Biological Sciences, University of Aberdeen, Aberdeen AB24 3FX, U.K.; ‡ Materials and Chemical Engineering, School of Engineering, University of Aberdeen, Aberdeen AB24 3FX, U.K.

**Keywords:** deterministic community assembly, bacteria, hydrocarbon degradation, ecosystem functional resilience, dispersion, diversity

## Abstract

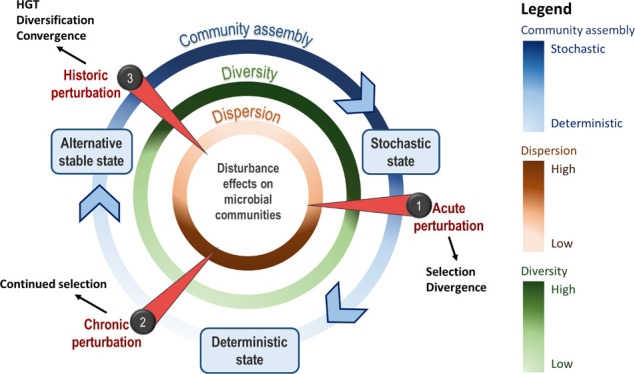

Acute environmental perturbations are reported to induce deterministic microbial community assembly, while it is hypothesized that chronic perturbations promote development of alternative stable states. Such acute or chronic perturbations strongly impact on the pre-adaptation capacity to the perturbation. To determine the importance of the level of microbial pre-adaptation and the community assembly processes following acute or chronic perturbations in the context of hydrocarbon contamination, a model system of pristine and polluted (hydrocarbon-contaminated) sediments was incubated in the absence or presence (discrete or repeated) of hydrocarbon amendment. The community structure of the pristine sediments changed significantly following acute perturbation, with selection of different phylotypes not initially detectable. Conversely, historically polluted sediments maintained the initial community structure, and the historical legacy effect of chronic pollution likely facilitated community stability. An alternative stable state was also reached in the pristine sediments following chronic perturbation, further demonstrating the existence of a legacy effect. Finally, ecosystem functional resilience was demonstrated through occurrence of hydrocarbon degradation by different communities in the tested sites, but the legacy effect of perturbation also strongly influenced the biotic response. This study therefore demonstrates the importance of perturbation chronicity on microbial community assembly processes and reveals ecosystem functional resilience following environmental perturbation.

## Abstract 2

Hydrocarbon perturbation chronicity affects microbial communities, with legacy effects in historically contaminated sediments and varied responses in pristine environments.

## Introduction

The microbial community structure is driven by many biological and environmental factors, and the underlying controlling mechanisms are referred to as community assembly processes. The microbial community structure is relatively stable over time, and community assembly theory defines two states. A deterministic state corresponds to a system situation fully determined by predictable parameter values and the initial conditions. In contrast, a stochastic state refers to a phase in which variables influencing the subsequent state of a system are determined by a certain level of unpredictability or randomness. Microbial communities play important roles in the biodegradation of environmental pollutants, including hydrocarbons in marine environments, necessitating increased understanding of microbial community assembly processes following environmental perturbations. In unperturbed, stable environments, community assembly is believed to be governed by stochastic processes and, based on neutral theory, is mediated by dispersal, drift, and speciation.^1^ In contrast, deterministic assembly is driven by contemporary natural or anthropogenic environmental perturbation, which induces selection of microbial traits, or exclusion of taxa, so that the community is better adapted to the new conditions.^2,3^ Deterministic selection is favored by increased intensity of environmental perturbation,^4,5^ but different responses have been reported. Different initial communities subjected to the same perturbation may converge to communities with similar phylogenetic composition^6^ or may diverge.^7−9^ Acute (usually intense and short-term, *e.g.*, hours/days) pollution is therefore likely to transform communities through deterministic selection, while chronic (ongoing, usually less intense than acute and long-term, *e.g.*, weeks) pollution can lead to a new stable state.^10^

Microbial community assembly processes are contingent on the nature of the perturbation and new environmental characteristics but are also influenced by previous community history^11,12^ and previous environmental disturbances. For example, historic chronic perturbation can have a prolonged impact on a community even after removal of the perturbation, termed a legacy effect.^11^ This effect may determine the ability of the community to adapt rapidly and track environmental change. Indeed, pre-conditioning of a community to a perturbation facilitates adaptation of the microbial community, through “memory” of historical perturbations.^6,13^ Changes in the community structure will influence the nature and rates of the microbial functions,^14−16^ providing alternative and potentially beneficial functions, such as biodegradation and remediation of a contaminated site,^17−20^ while maintaining ecosystem functional resilience within the global community^21^ (with ecosystem functional resilience referring to the ability of a community to continue to carry out a specific function due to the existence of functional redundancy^22^).

Despite the wealth of research on microbial community assembly processes (see^23^ for a review), several important questions remain: (a) Does chronic perturbation affect community assembly processes? (b) Does pre-conditioning of a community buffer chronic perturbations? (c) Following an initial acute perturbation, does a secondary, identical perturbation maintain the newly adapted community structure or cause additional modifications? (d) Is ecosystem functional resilience important following environmental perturbation? Answering these questions will obviously depend on the nature, strength, and repeatability of perturbations and the history of the sites analyzed. In this study, we investigated these questions by focusing on hydrocarbon (HC) pollution in marine sediments. Perturbation was achieved by supplementation of sediment with phenanthrene, a model three-ring polyaromatic HC persistently detected in HC-perturbed environments and a potential carcinogen.^24^ HC pollution is indeed a common and global environmental perturbation, and there is considerable evidence of rapid changes in the microbial community structure following acute HC pollution.^25−29^ HC degradation is well documented^30^ and is performed by phylogenetically and functionally diverse microorganisms that can degrade identical HCs at different rates.^31−33^ HC degradation therefore allows study of community assembly processes and ecosystem functional resilience of natural communities in an important ecological and economic context.

**Figure 1:**
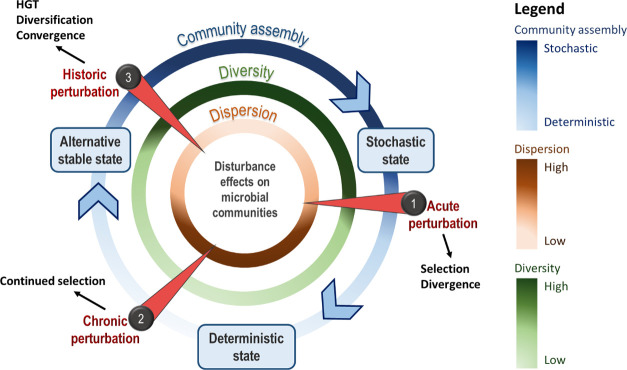
Conceptual model illustrating the effects of acute and chronic environmental disturbance on microbial community assembly processes. (1) Acute perturbations induce deterministic assembly where niche-specific specialists are selected resulting in decreased community diversity. Due to interspecies interactions such as competition, cooperation, and succession, distinct communities under the same perturbation will diverge phylogenetically resulting in increased community dispersion. (2) Continued (chronic) perturbation will maintain this deterministic state with continued selection of specialists until an alternative stable state is reached. (3) Perturbation on a decadal, or longer, scale will cause deterministic processes to be overruled by random stochastic processes such as dispersal. A permanent change in the environment may promote community diversification and a cumulative increase in horizontal gene transfer (HGT) events allowing the community to adapt evolutionarily and thrive. This results in restoration of higher microbial diversity and a reduction in community dispersion.

The main research objective was, therefore, to understand the impact of both chronic and acute perturbations on microbial community assembly processes in the context of hydrocarbon contamination. Several sediments from both estuarine and marine environments were selected to represent a gradient of HC pollution, from non-contaminated (“pristine”, hereafter) to chronically contaminated (“polluted”, hereafter) sites. These sites were exposed to an acute disturbance (HC addition) to test the following hypotheses as illustrated in a conceptual model in Figure 1: (1) exposure of pristine sediments to HC will induce deterministic microbial community assembly through strong selection of HC-degrading microorganisms, resulting in community dispersion (*i.e.,* increased variation of community composition); (2) addition of HC to both polluted and HC-amended pristine sediments will sustain deterministic assembly processes until an alternative stable state is reached, which is then primed to respond to HC contamination; and (3) permanent disturbance results in a stochastic state through community diversification, allowing communities to adapt to and function in the new environment. In addition, it is proposed that ecosystem functional resilience for HC degradation is similar across replicates within each site, regardless of community composition.

## Materials and Methods

### Site Sampling and Microcosm Setup

To test the effect of chronic environmental perturbation on microbial community assembly, we used databases and literature searches to identify 10 sites in the United Kingdom that are well-known for their higher levels of pollution (Figure S1), providing a gradient of the total petroleum hydrocarbon (TPH) concentration (see Figure S2 and Table S1 for details).^34,35^ For each site, five surficial sediments (0–2 cm) were sampled, combined, homogenized, and stored at 5 ± 2 °C for 8 days, which was similar to measure *in situ* temperatures. The TPH concentrations in sediments were analyzed by QTSE Environmental Ltd, owned by DETS Ltd, using a GC-MS method according to MERTS and UKAS standards. Other physico-chemical properties of the sites, such as total organic carbon levels, were not measured, and it is acknowledged that these can influence the behavior and biodegradation of hydrocarbons.

**Figure 2:**
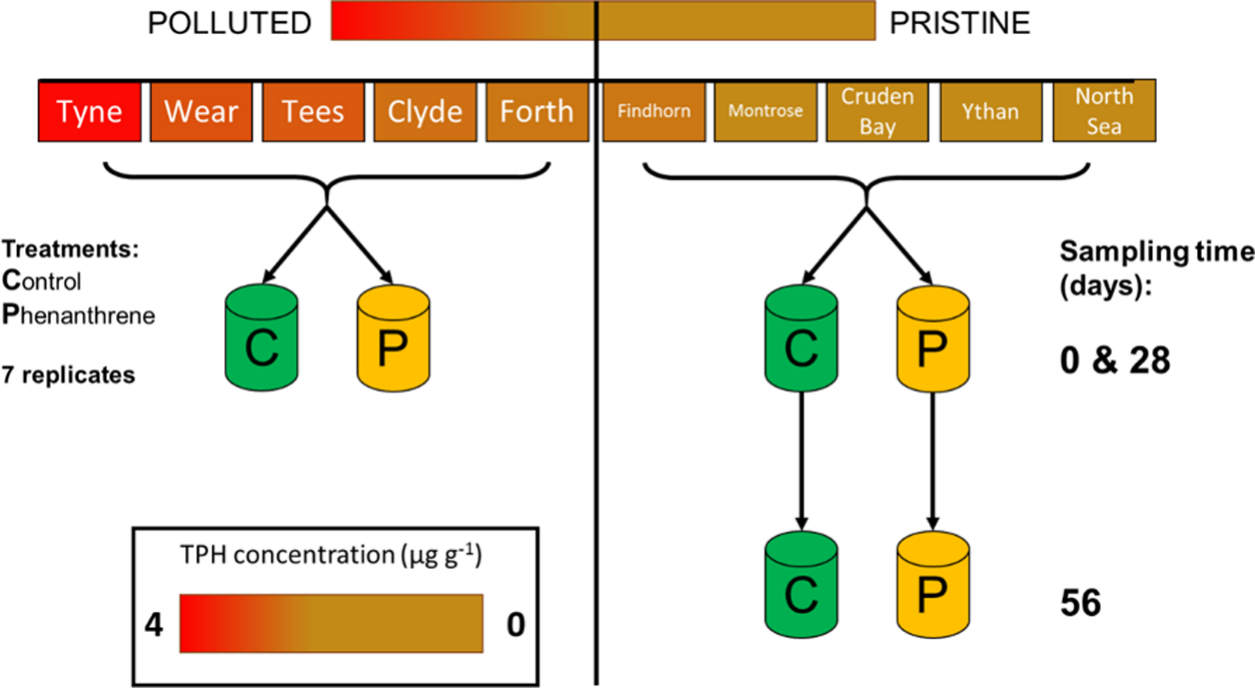
Schematic of the experimental design. Five polluted sites (Tyne, Wear, Tees, Clyde, and Forth) and five pristine sites (Findhorn, Montrose, Cruden Bay, Ythan, and the North Sea) were sampled based on pollution history using literature and database resources, and their classification as “polluted” and “pristine” was based on the measured total petroleum hydrocarbon (TPH) concentration. The TPH concentration within each site prior to incubation is shown as a color gradient from highest (red) to lowest (light brown). Each site was treated with phenanthrene (P; yellow) or left untreated as a control (C; green), with seven replicates for each treatment. Pristine sites were also amended with additional phenanthrene on day 28 to simulate a chronic perturbation.

The 10 selected sites represent a gradient of HC pollution, from non-contaminated (“pristine,” hereafter) to chronically contaminated (“polluted,” hereafter) sites (Figure S2). Although contamination at all sites was lower than that in reported heavily polluted sites, TPH levels were grouped into three classes: below detection at four sites (Montrose, Cruden Bay, Ythan, and the North Sea), intermediate at three sites (Clyde, Forth, and Findhorn), and relatively high at three sites (Tyne, Wear, and Tees). Despite such a tight gradient, the sites were sufficient to test our predictions, and we classified the 10 sites as polluted and pristine sites based on measures of the TPH concentration in sediments and on the literature as defined in Figure 2 for all statistical analyses.

Seven replicated microcosms were established containing the untreated control (C) and phenanthrene-treated (P) sediment from each site (see the sample coding in Supporting Information 1 and the experimental design in Figure 2). These 140 microcosms were incubated for 28 days, and the 70 microcosms established from the pristine sites were supplemented with the same amount of phenanthrene as initially and incubated for a further 28 days to stimulate chronic perturbation.

Phenanthrene was added to microcosms as described previously.^36^ Briefly, phenanthrene was weighed into autoclave-sterilized (121 °C at 100 MPa for 21 min) 60 mL vials to give a final concentration of 0.1% (w/w) within bulk sediment. Phenanthrene was dissolved by adding 2 mL of acetone (HPLC grade; Sigma-Aldrich, UK) to vials and mixed with 2 g of site-specific sediment until homogeneous. The same procedure was adopted for control microcosms without phenanthrene addition. Following evaporation of acetone for 24 h, 18 g of sediment was added to each vial, and vials were loosely screw-capped and incubated at 20 °C with agitation at 75 rpm. The vials were opened every 3–4 days in a sterile environment to exchange airspace. Sediment samples (∼1 g) for molecular analysis (nucleic acid extraction and microbial community analysis) were taken at the surface of the vials at days 0 and 28 for all samples and day 56 for all pristine sites (both control and phenanthrene-treated) and stored at −80 °C until further analysis. Microcosms were destructively sampled at the end of incubation for phenanthrene analysis.

To ensure incubations still contained sufficient levels of phenanthrene to promote a microbial response at the end of incubations and represent a perturbation over the course of the incubation, an additional set of triplicate microcosms was established and destructively sampled after 21 days. Moreover, a further separate set of triplicate microcosms was established for abiotic degradation controls (such as pH, temperature, or UV that can possibly degrade phenanthrene) using Tyndallized sediment (autoclaved three times over 3 consecutive days).

Microcosm sediment results are referred to as sites hereafter, with control and phenanthrene-treated representing microcosms without or with phenanthrene supplementation, respectively.

### DNA Extraction, Sequencing, and Processing

Total genomic DNA was extracted from 0.4 g of sediment using the FastDNA SPIN Kit for Soil and FastPrep-24 instrument (both MP Biomedicals, Cambridge, UK), according to the manufacturer’s instructions. DNA extracts were quantified using a spectrophotometer (NanoDrop ND-1000) and then stored at −80 °C until further analysis.

The universal bacterial and archaeal V4 regions of the 16S rRNA gene were amplified with the primer set 515F/806R^37^ using the KAPA Hi-Fidelity enzyme (Roche Diagnostics, UK). Prior to MiSeq Illumina sequencing, PCR-amplified sequences were cleaned using AMPure XP beads (Beckman Coulter), and PCR-indexing was performed using the Nextera XT Index kit according to the manufacturer’s protocol. Following further cleaning, library quantification, normalization, and pooling of samples were performed prior to paired-end MiSeq sequencing. Two runs of amplicon sequencing were performed, using the V3 (2 × 300 bp) chemistry (CGEBM, University of Aberdeen, Aberdeen) and the V2 (2 × 250 bp) chemistry (NCIMB Ltd, Aberdeen) to accommodate all the samples. Forward and reverse reads were screened for a phred quality score greater than 30 and minimum length of 200 bp using Trim Galore v 0.5.^38^ All sequences were truncated to 200 bp using vsearch v 2.8 to optimize sequencing assembly.^39,40^ Sequence processing and assembly were performed using Mothur software v 1.39.5^41^ on the Maxwell high performance computing cluster (University of Aberdeen). Using default parameters in Mothur, sequences were aligned against the SILVA reference database v132,^42^ chimeras were detected and removed using vsearch, and singletons were also removed. OTUs were clustered at 97% similarity using the “opti” method, and the taxonomy was assigned using the SILVA reference database.

### Phenanthrene Extraction and Quantification

Phenanthrene was extracted from microcosm sediment to determine the microbial degradation potential. Prior to extraction, sediments were spiked with 100 μL of a surrogate standard solution of pristane in dichloromethane (20 μL mL^–1^ each) to assess extraction efficiency. Anhydrous sodium sulphate (5 g) was added to the samples to remove interstitial water. Sediments were sequentially extracted thrice with 10 mL of dichloromethane by ultra-sonication for 10 min. Extracts were combined and centrifuged at 3000 rpm for 10 min to remove suspended materials. The dichloromethane/phenanthrene analyte was then transferred to PTFE-capped gas chromatography vials for analysis by gas chromatography (Varian CP3800 with a 30 m Zebron ZB-50 column) fitted with a flame ionization detector (GC-FID). An internal standard (20 μL mL^–1^ pentadecane in dichloromethane) was spiked into extracts immediately prior injection to account for injection error. Nitrogen was used as the carrier gas at a constant flow rate of 0.84 mL min^–1^. One μL of the sample was injected with a split ratio of 10:1. The injector and detector temperatures were 330 °C; initial oven temperature was 50 °C with a 3 min hold and then increased at 10 °C min^–1^ to 110 °C, followed by an increase to 200 °C at 5 °C min^–1^ with a 12 min hold. Temperature was increased finally to 300 °C at 20 °C min^–1^ and held for 6 min. The extraction efficiency was 86.1 ± 2.1% based on surrogate standard data. A six-point calibration curve was generated for phenanthrene to determine gas chromatography linearity and retention factor responses (see ref (43) for more detail).

### Statistical Analysis

All analyses were performed in R v 4.0.3,^44^ and figures were produced using the *cowplot* (https://cran.r-project.org/web/packages/cowplot/index.html) and *ggplot2*(45) packages.

Standard measures of alpha diversity of 16S rRNA genes (Shannon and Pielou indexes) were estimated using the *vegan* package.^46^ Differences in alpha diversity between treatments were examined by fitting linear mixed effects models (LMM) using the nlme package (v 3.1)^47^ where we included fixed effects of treatment, time, and an indicator variable HC to denote polluted and pristine sites (as defined in Figure 2). We included a three-way interaction between these variables (and all associated two-way interactions) to determine whether alpha diversity changed over time, whether differences were dependent on treatment (control and phenanthrene), and whether these differences were consistent between polluted and pristine sites. We also included a random effect of the site using a random effect structure that allowed for sites to respond differently over time. The optimal random effect structure was determined using likelihood ratio tests (LRTs) comparing nested models fitted using restricted maximum likelihood (REML). The fixed effects were tested using LRT-comparing nested models fitted using maximum likelihood (ML). The final models also included a variance covariate (using the varIdent function) to estimate a separate variance for each time period and/or for each site. All final models were refitted using REML, and standard diagnostic plots of residuals were used to assess modeling assumptions. Subsequent pairwise comparisons of alpha diversity between relevant treatment groups were performed using the emmeans package (v 1.6)^48^ and p-values adjusted to control for the type I error rate using Tukey’s method. Due to the unbalanced experimental design, this approach was applied on all pristine and polluted sites over 28 days (days 0 and 28) (see details in Supporting Information statistics 1 and 3) and on the pristine sites only over 56 days (days 0, 28, and 56) (see details in Supporting Information statistics 2 and 4).

Beta diversity was estimated using the vegdist function^49^ with default parameters used in conjunction with the Bray–Curtis distance metric, and ordination was plotted by performing nonmetric multi-dimensional scaling using the function metaMDS.^50^ Ellipses (95% confidence) highlighting clustering of site-specific communities were drawn using the function ordiellipse. Differences in the Bray–Curtis distance metrics over time, between the site category (polluted or pristine), and treatments were analyzed with PERMANOVA using the vegan function adonis.^49^ Permutations were constrained by site (see details in Supporting Information statistic 5). Community dispersion was estimated with the function betadisper, which plots the data coordinates within a principal coordinates analysis (PCoA) space and determines the centroid of a defined set of samples (with the replicates being grouped by site category, treatment, and time combination). The Euclidean distance is then measured from each group to the centroid, providing a measure of multivariate dispersion between replicates. A linear mixed effects modeling approach similar to the alpha-diversity analysis was then used to identify differences between treatment, site category, and time. Models were fitted on all pristine and polluted sites over 28 days (see details in Supporting Information statistic 6) and on the pristine sites only over 56 days (see details in Supporting Information statistic 7).

Finally, a phylogenetic clustering model (Beta Nearest Taxon Index: βNTI) was applied to this dataset to quantify potential deterministic processes. This model assumes the presence of a phylogenetic signal in the dataset. Each sample was rarefied to 500 reads, and the 1000 most abundant OTUs were selected. The resulting sequences were aligned using MAFFT v 7.453,^51^ and a phylogenetic tree of the resulting OTUs was constructed using IQ-TREE v 1.6.12.^52^ The phylogenetic signal was then tested using the phylogenetic mantel correlogram provided by the function phylosignal from the package picante^53^ (see details in Supporting Information statistic 8).

Phenanthrene degradation over time was estimated for polluted sites at day 28 and for pristine sites at day 56 (due to the requirement of destructive sampling for phenanthrene quantification). To account for the difference in the time period, the initial phenanthrene concentration was supplemented twice in the pristine sites compared to the polluted sites. Therefore, we calculated the percentage degradation [(start concentration – end concentration)/start concentration] instead of using the final concentration. Similar to the alpha-diversity and dispersion analysis, we used a linear mixed effects model to analyze phenanthrene degradation and included the treatment, time, and site category (HC) as fixed effects, a three-way interaction between these variables (and all associated two-way interactions) and a site random effect to account for between site variability (see details in Supporting Information statistic 9).

## Results

### Microbial Diversity and Community Structure

The 16S rRNA MiSeq sequencing approach yielded an average of 48,663 reads per sample [±1,143 standard deviation (SD)]. Five samples (out of 350) were omitted due to low read depth (TS_C_1_3, YT_C_1_3, WE_P_0_4, CL_P_0_1, and FH_C_1_2). Samples were then rarefied to 9,000 reads (the lowest read depth in all samples) before further analysis.

**Figure 3:**
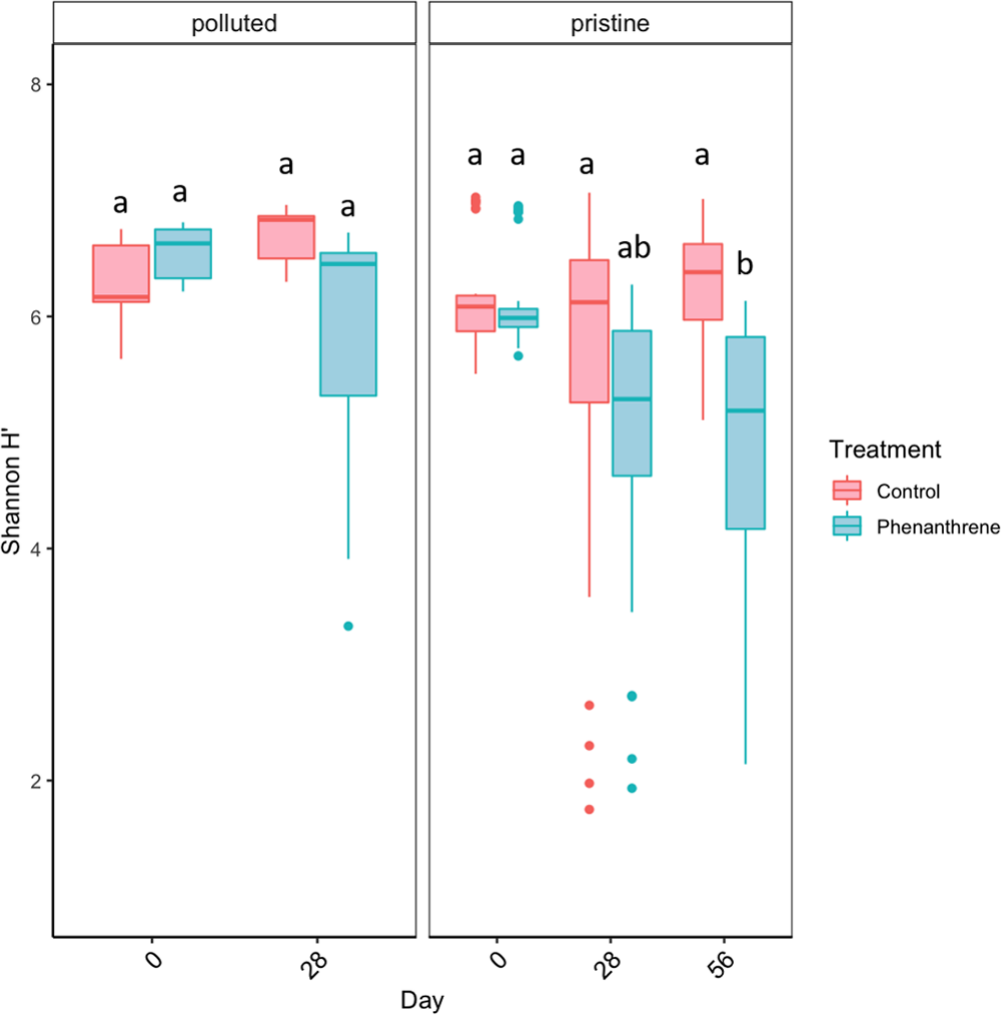
Estimated alpha diversity (Shannon index) across all the pristine and polluted sites in control and phenanthrene-treated communities over time; only the pristine communities were incubated for 56 days. Letters indicate significant differences and are based on statistical analyses performed over 28 days for the polluted sites (see Supporting Information Statistic 1) and over 56 days for the pristine sites (see Supporting Information Statistic 3).

Shannon diversity (*H*′) estimates (Figure 3) differed between treatments (control or phenanthrene), and this difference was different over time (over the 28 days period) and whether the samples came from a polluted or pristine site (Supporting Information Statistic 1: LMM; three-way interaction between the treatment, time, and site category; *F-*value = 5.8033 and *P*-value = 0.0167). These Shannon estimates were initially similar between all control sites (mean 6.25 ± 0.52 SD) (LMM contrast pristine-polluted, *P*-value = 0.9970) and remained constant during incubation over 28 days for the polluted sites (Supporting Information Statistic 1: LMM contrast day 0-day 28, *P*-value = 0.2906) and over 56 days for the pristine sites (Supporting Information Statistic 2: LMM contrast day 0-day 56, *P*-value = 0.8303). In phenanthrene-treated communities, diversity significantly decreased over time in pristine sites (Supporting Information Statistic 2: LMM contrast day 0-day 56, difference = −1.2508, *P*-value < 0.0001) but not in the polluted sites (LMM contrast day 0-day 28, *P*-value = 0.0871). Evenness (estimated using Pielou’s J index) followed a similar pattern (Figure S3; Supporting Information Statistics 3 and 4).

**Figure 4:**
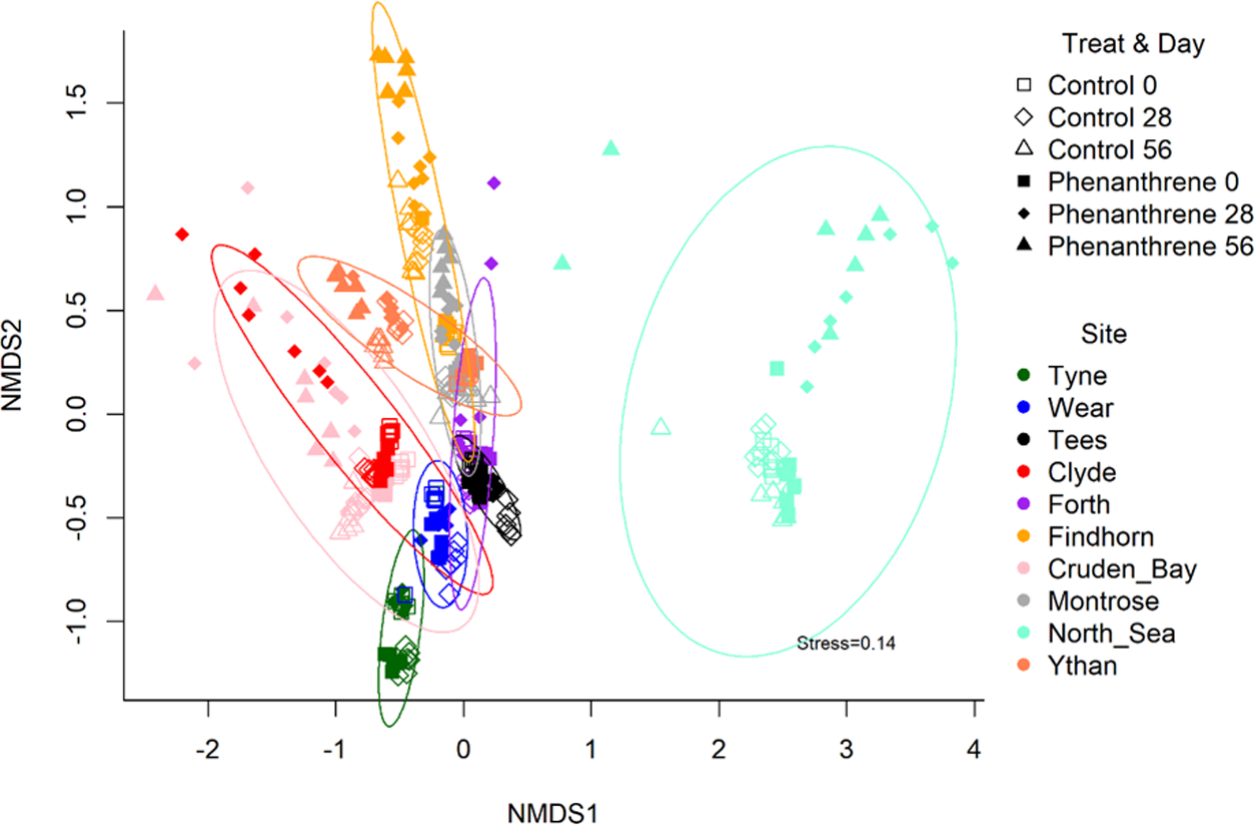
Ordination (non-metric multi-dimensional scaling; nMDS) of all sites, treatments, and time points based on the dissimilarity of community composition between sites over time. Ellipses indicate grouping of microbial communities per site (encompassing all treatments and time) at the 95% confidence interval. The order of the sites in the legend corresponds to their initial level of contamination (from highest to lowest) as presented in Figure 2.

**Figure 5:**
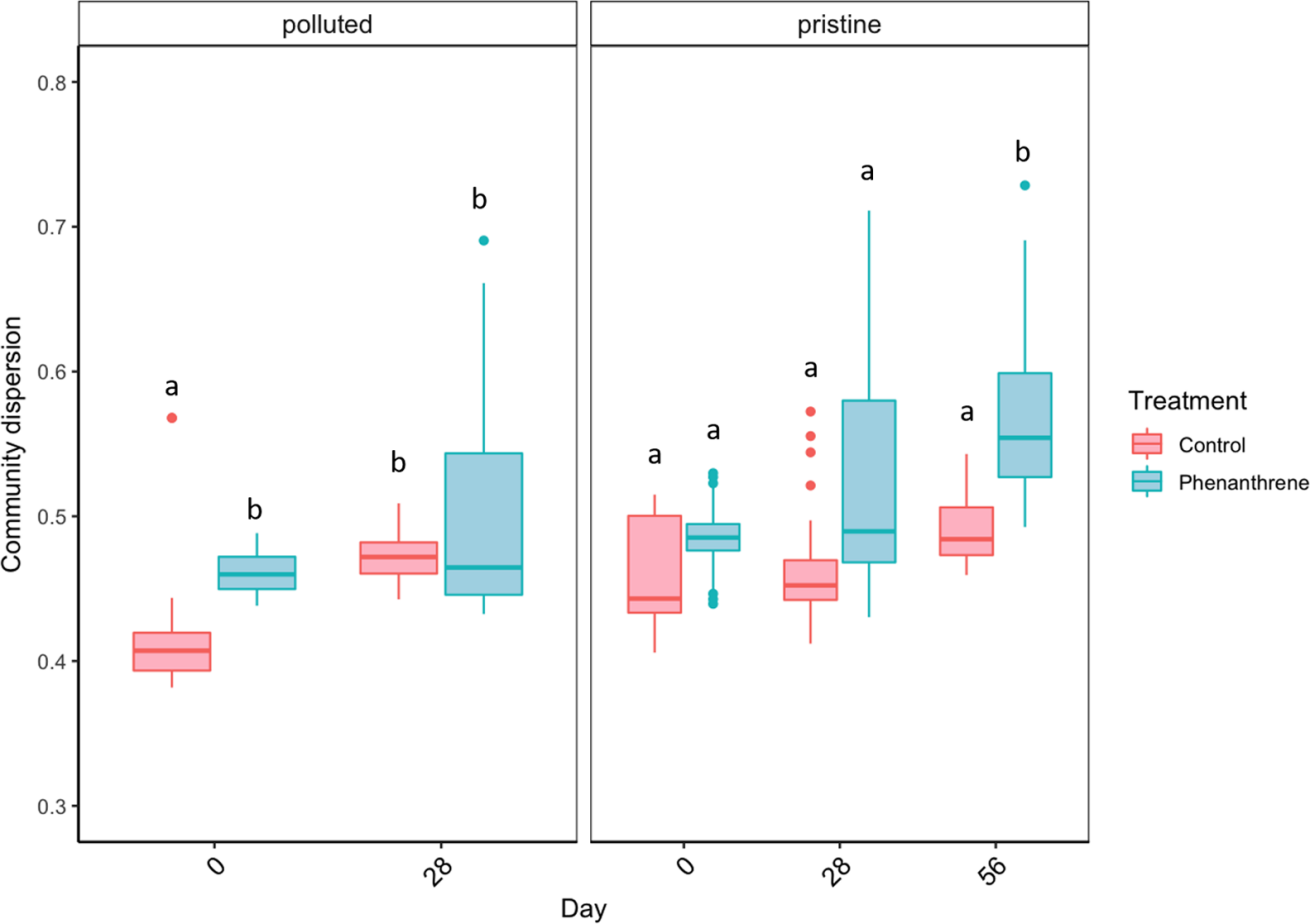
Estimated degree of community dispersion within the pristine and polluted sites in control and phenanthrene-treated communities over time; only the pristine communities were incubated for 56 days. This index is calculated as the Euclidean distance in the principal coordinate space between each sample replicate and its respective group centroid. Letters indicate significant differences and are based on statistical analyses performed over 28 days for the polluted sites (see Supporting Information Statistic 6) and over 56 days for the pristine sites (see Supporting Information Statistic 7).

Microbial community composition was significantly different between control and phenanthrene-treated samples, and these differences were dependent on time and whether samples were from pristine or polluted sites (Figures 4, S4, S5; Supporting Information Statistic 5: adonis, *P*-value < 0.0001). Variation in the microbial community structure was also analyzed *via* an index of microbial community dispersion between replicates, with replicates being grouped by the site category (pristine or polluted), treatment, and time combination. Microbial dispersion differed between treatments (control or phenanthrene), and this difference differed over time (over the 28 days period) and depended on the sample origin (whether the samples came from a polluted or pristine site) (Figure 5; Supporting Information Statistic 6: LMM; significant three-way interaction between the treatment, time, and site category; *F*-value = 10.9251 and *P*-value = 0.001). In the absence of phenanthrene, the mean dispersion remained constant over 56 days for the pristine sites (Supporting Information Statistic 7: LMM contrast day 0-day 56, *P*-value = 0.3899) but increased over the 28 days for the polluted sites (Supporting Information Statistic 6: LMM contrast day 0-day 28, *P*-value = 0.0456). In the presence of phenanthrene, the mean dispersion remained constant over 28 days for both the pristine and polluted sites (Supporting Information Statistic 6: *P*-value = 0.1087 and 0.1396, respectively), but the mean dispersion increased in the second incubation period (between days 28 and 56) for the pristine sites (Supporting Information Statistic 7: LMM contrast day 28-day 56, *P*-value = 0.0494) resulting in a continuous community dispersion for those sites over the whole incubation (Supporting Information Statistic 7: LMM contrast day 0-day 56, *P*-value = 0.0006).

To quantify deterministic processes involved in the diversity differences, we aimed to apply a phylogenetic clustering model (Beta Nearest Taxon Index: βNTI) to this dataset. This approach has been previously applied to different datasets following identification of a phylogenetic signal, which is the statistical tendency of related phylotypes to share more trait values than random phylotypes from the same tree, due to their phylogenetic relationship.^10,54^ However, analysis of the phylogenetic mantel correlogram in this dataset indicated an absence of a significant phylogenetic signal (Figure S6), which prevented application of this approach.

### Community Composition

The heatmap representing the relative abundance of the 20 most abundant families of the total community (based on the 16S rRNA gene) indicates that communities were not frequently strongly dominated by a single family (Table 1). Bacteria dominated phenanthrene-treated sediments at day 0 in all sites except the North Sea, which contained 24% of archaea of the family *Nitrosopumilaceae* (Table 1). However, it is recognized that there are known biases with the universal primer pair used here, including underestimation of SAR11 and Thaumarchaeota/Crenarchaeota.^55^ The most common bacterial phyla in control sediments were Actinobacteria, Bacteroidetes, Chloroflexi, Planctomycetes, and Proteobacteria (mainly α, β, and γ).

**Table 1:**
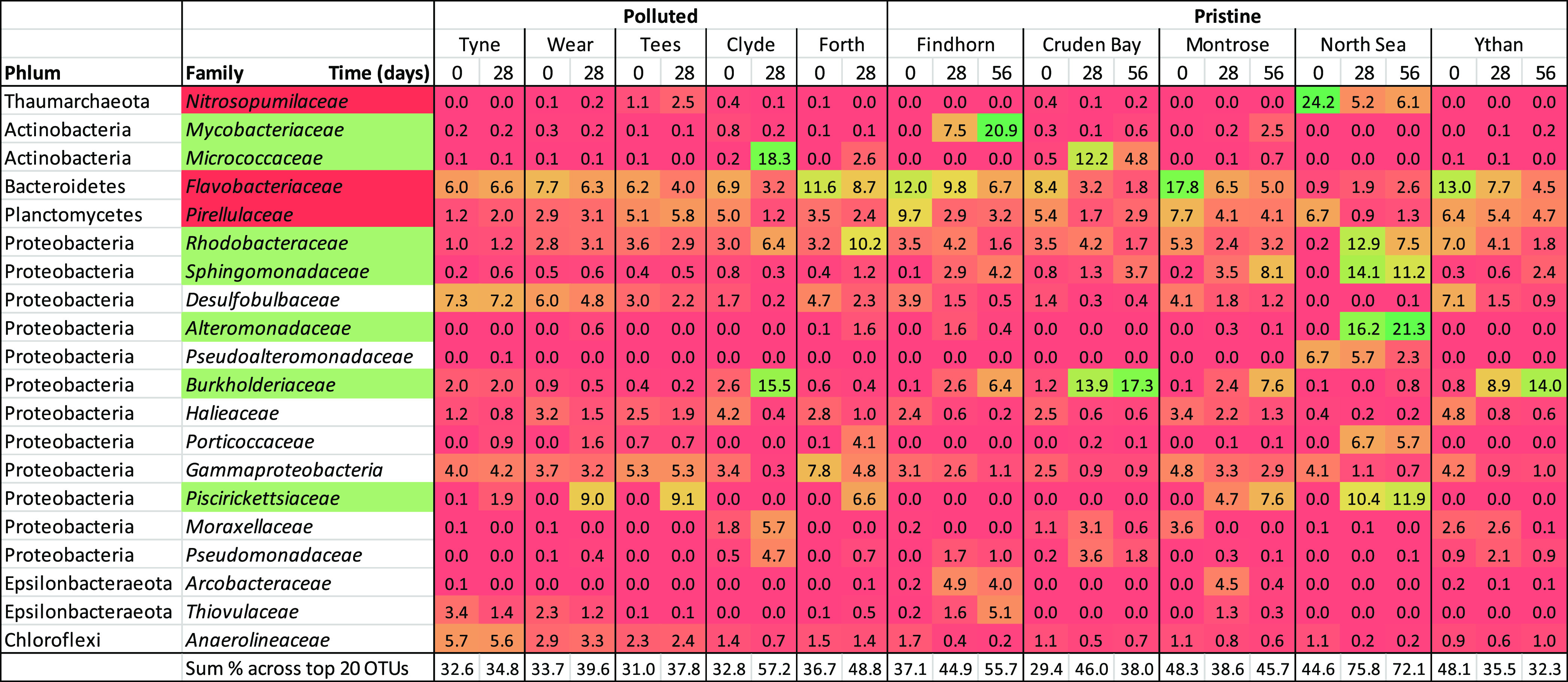
Heatmap Representing the Relative Abundances (as a Percentage of the Whole Community) of the 20 Most Abundant Taxa (Across all Sites) at Phylum and Family Levels in Phenanthrene-Treated Communities^a^

a Relative abundances were estimated based on 16S rRNA gene sequences in seven replicates per sites (except for Wear day 0 and Clyde day 0, which were based on six replicates). The color range (red to green) represents percentage abundance (low to high, respectively). Taxa that were initially abundant at <0.1% and increased to >10% are highlighted in green, and taxa that were initially >10% and decreased over time are highlighted in red. Standard deviations are presented in Table S2.

Among major community changes observed over time, the relative abundance of a diverse range of 10 families changed by >10% over time in at least one site (Table 1). Several bacterial families, *e.g.*, *Burkholderiaceae*, *Rhodobacteraceae,* and *Piscirickettsiaceae*, were selected in several sites. In contrast, the relative abundance of several families (*e.g.*, *Flavobacteriaceae*, *Pirellulaceae,* and *Nitrosopumilaceae*) decreased during incubation with phenanthrene, these changes being more prominent in pristine sites (Table 1).

### Phenanthrene Biodegradation

**Figure 6:**
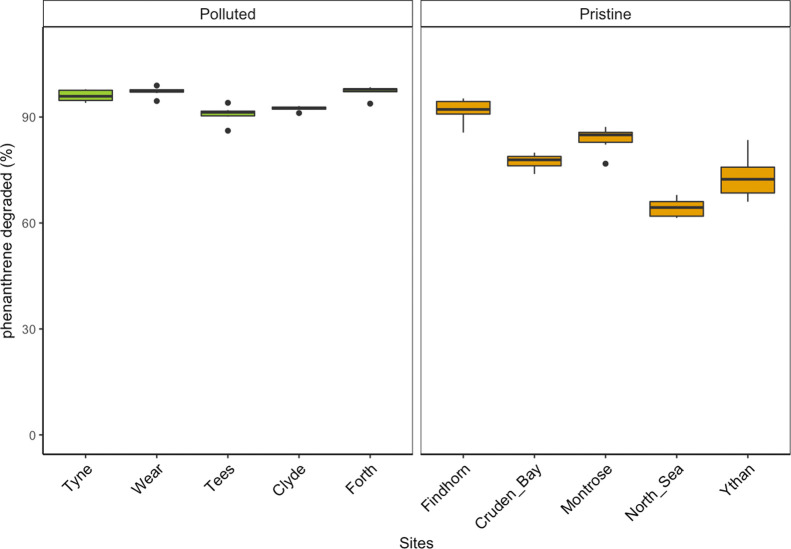
Biotic degradation of phenanthrene after incubation for 28 days in the polluted sites and 56 days in the pristine sites, which accounts for additional phenanthrene addition. Degradation was calculated based on the remaining proportion of the supplemented phenanthrene after incubation.

In order to estimate as accurately as possible the level of phenanthrene degradation, we ensured that phenanthrene was present in microcosms throughout the incubation period and estimated that 9 ± 3 and 28 ± 6% of the total added HC remained at day 21 within polluted and pristine sediments, respectively. In addition, most phenanthrene degradation was biotic, as <5% degradation occurred in the sterilized control microcosms (*n* = 30) over the entire incubation period. After incubation, phenanthrene degradation was greater in polluted than pristine sediments (95 vs 78%) (Figure 6; Supporting Information Statistic 9: LMM; *p* < 0.001), suggesting that pre-exposure facilitates degradation ability following contaminant exposure. Low degradation variability between replicates (Figure 6) contrasted with the high community dispersion (Figure 5) and high variability of dominant taxa (Tables 1 and S2).

## Discussion

Determining the impact of environmental perturbation on microbial community assembly provides insights into community resistance, resilience, ecosystem functional resilience, and ecosystem processes.^22,56−58^ In this study, we demonstrated that acute environmental change influenced the microbial community structure and ecosystem function differently, depending on the frequency of perturbation and the level of the historical legacy. Microbial communities from chronically perturbed sediments were more resistant to acute environmental change, whereas selection of specific microbes in non-perturbed sediments caused significant changes in the community structure. The underlying community assembly processes in both scenarios relate to the conceptual model (Figure 1), which proposes that a shift from a stochastic to a deterministic state corresponds to a decrease in diversity and an increase in community dispersion. This model does not consider the ecosystem function of the microbial communities, as functional redundancy will be highly dependent on community composition.

### Effect of Disturbance on Microbial Diversity, Community Structure, and Community Assembly Processes

Initial microbial community diversity was similar across locations between pristine and polluted sites, regardless of perturbation history (Figure 3; Supporting Information statistic 1). This was surprising, as several studies report reduced biodiversity in sediments subjected to environmental perturbations,^59−61^ but this could be explained by the relatively low level of contemporary contamination in the selected contaminated sites of our study. It is assumed that sediments used in this study which were subject to historic perturbation of 10–100s of years led to a stochastic state through events such as adaptive evolution through horizontal gene transfer,^62^ which is well documented in HC-degrading organisms (see^63^ for a review). Long-term environmental pressure is also known to promote community diversification of well-adapted phylotypes.^64^ The occurrence of these phylotypes in the different sites allows their putative classification as specialists and generalists based on their classical ecological definitions,^65^ with generalists being more geographically widespread than specialists but performing fewer ecosystem functions. Although our dataset does not allow clear distinction between specialists and generalists (in particular due to the relatively limited number of sites), several phylotypes affiliated to families known to degrade HCs were detected in chronically contaminated sediments, such as *Burkholderiaceae*, *Rhodobacteraceae,* and *Piscirickettsiaceae* (Table 1). This suggests the selection of habitat specialists under such conditions (see the “Selection of Hydrocarbon-Degrading Communities and Ecosystem Functional Resilience” section for more details). In addition, a more holistic characterization of specialists and generalists would require determination of the physiological traits of putative specialists.

Phenanthrene addition significantly decreased alpha diversity of microbial communities in pristine sites during the incubation period (Figure 3). Addition of HCs has frequently been reported to decrease total bacterial diversity,^61,66^ while the impacts of oil addition on archaeal communities are contradictory, with a decrease and increase in archaeal diversity observed in beach sand microcosms^67^ and water column samples,^68^ respectively. These changes are probably due to selection and growth of microbial communities capable of oil degradation, although this is based on relative abundance data, not quantitative abundance of each taxon. In addition, perturbation of pristine sediments in the present study led to microbial community dispersion related to a broader phylogenetic content (Figure 5), that supports community restructuring and potential deterministic selection of different habitat specialists. Incubation of polluted sites constrained microbial community dispersion (Figure 5), suggesting maintenance of a stable community mediated by stochastic processes with continued selection of habitat specialists. Although such an approach could not be applied in our study, quantification of the proportion of deterministic and stochastic processes in microbial systems using null models and associated indices, such as the β-nearest taxon index (βNTI),^54^ previously revealed that deterministic assembly was associated with environmental changes in non-perturbed environments.^3^

Inclusion of a relatively large number of replicates for each site and multiple sites enabled assessment of dispersion of community composition following disturbance. This approach provided evidence for the hypothesis that pristine sediment communities diverge from their initial composition following phenanthrene amendment due to heterogeneous deterministic selection. Such deterministic selection has also been reported in sediment-water communities,^69^ with several potential selection mechanisms, both following an oil perturbation in marine sediments or perturbations in soil (*e.g.*, drought, fertilizer amendment, ploughing, etc). First, interspecies interactions result in variable responses due to complex dynamics between microbial communities and their specific environments.^70^ Second, niche differentiation and specialization can result in co-occurrence of phylogenetically different but functionally redundant taxa.^71^ Third, competition for resources may result in non-specific selection of taxa if microorganisms have similar resource affinities and growth rates.^72^

### Influence of Perturbation Chronicity on Microbial Community Assembly

All sites in this study had relatively low levels of contamination compared to the previous literature, of which three sites presented higher levels; the distinction between high and moderate contamination is relatively arbitrary due to the skewed gradient of contamination toward lower concentrations (Figure S2). As expected, the initial community structure was not fully controlled by hydrocarbon contamination, with some polluted or pristine sites presenting similar composition (*e.g.,* Clyde and Cruden Bay, Figure 4), probably due to the influence of other biotic and abiotic factors. Visual analysis of temporal changes in community composition (Figure 4) provided evidence for the hypothesis that communities pre-adapted to a specific perturbation were primed and became resistant to that environmental disturbance. In addition, in the polluted sites, community composition was maintained throughout additional perturbation (Figure 4, Table 1), and both community diversity and dispersion remained unchanged following perturbation (Figures 3 and 5). Such maintenance of community composition, despite environmental disturbance, can be explained by community history, which is often a better predictor of community assembly than contemporary environmental conditions.^12^ Pre-conditioning a community to a new habitat results in predictable and reproducible community assembly.^6^ In particular, pre-exposure of microbial communities to HCs is known to prime the microbial response.^13,73^ Microbial communities within the Gulf of Mexico were believed to be pre-conditioned to HC exposure from natural crude oil seeps, which was postulated as a major factor for the rapid response of water column microbial communities to HC influx following the *Deepwater Horizon* oil spill.^27^

The responses of phenanthrene-treated polluted and both sets of phenanthrene-treated pristine sites can theoretically be fitted to a recently described species-sorting model,^11^ which determines the impact of legacy effects on the community response to environmental perturbation. This model considers four different scenarios: (1) no legacy effect, (2) transient legacy effect, (3) persistent legacy effect, and (4) mixed scenario.^11^ In this study, polluted sites were subjected to a long-lasting legacy of exposure to HCs and other pollutants, resulting in limited community composition shifts following perturbation (scenario 3). Conversely, the pristine sites displayed a gradual community shift following perturbation over the two periods of incubation with evidence of community shifts *via* species sorting, representing a transient legacy effect and maintenance of an alternative state (scenario 2).

### Selection of Hydrocarbon-Degrading Communities and Ecosystem Functional Resilience

Pristine communities perturbed with phenanthrene promoted preferential selection of families with known HC-degrading members across different geographical sites (*e.g.,Burkholderiaceae*, *Rhodobacteraceae,* and *Piscirickettsiaceae*), despite differences in initial community composition (Table 1).^74−76^ Selection of these families induced significant community changes, which are frequently observed in HC contamination studies,^74−76^ as contemporary environmental heterogeneity selects for niche-specific organisms. Selection of multiple microbial families upon addition of a single HC source is common,^72,77−79^ as distinct bacterial families are able to coexist. For example, strong selection of *Burkholderiaceae* at several sites suggests their prominent role in phenanthrene degradation as previously demonstrated by stable isotope probing.^80^ Similarly, members of the *Rhodobacteraceae* family were also retrieved in several phenanthrene-treated communities, probably due to their high polyaromatic hydrocarbon-degradation potential.^81^ Finally, *Piscirickettsiaceae’s* (specifically the genus *Cycloclasticus*) relative abundance increased following phenanthrene addition (from <0.1% initially to 6–12%), which reflects its capacity to respond rapidly to polyaromatic hydrocarbon addition.^82−84^ Although no absolute abundance was estimated in the present study, one may expect selective growth of these taxa rather than death of the other taxa, and specific selection of functionally relevant taxa from the rare biosphere has been discussed previously.^85−87^

Generic microbial functions such as respiration and biomass production are believed to be more redundant than specialized functions such as HC degradation,^88^ given the specificity of the genes and enzymes required for metabolism of specific HC structures (see ref (37) for examples). Following perturbation, phylogenetic diversity of HC-degrading organisms is known to increase, leading to a higher HC-degrading capability.^89,90^ Perturbation of sediment communities in this study resulted in varying levels of biotic phenanthrene degradation between polluted and pristine sites (Figure 6). For the communities who have reached a stable state following perturbation (*e.g.,* the polluted sites), phenanthrene degradation was high and consistent across all sites despite different community structures (Figure 6, Table 1). This ecosystem functional resilience between replicated disturbed communities suggests functional similarity as previously suggested^22^ and therefore supports previous evidence for functional redundancy within HC-degrading systems^91−94^ and novel evidence of functional similarity in such systems. Although the taxonomic level responsible for this ecosystem functional resilience should be further examined, the importance of drivers other than community composition such as abundance and activity of competent contaminant degraders or environmental conditions in the sediment would also require further investigation as both can influence rates of phenanthrene degradation. For example, higher residual levels of a contaminant could remain in organic-rich sediments due to sorption and reduced bioavailability, even in the presence of organisms with similar metabolic capabilities. To summarize, this study reinforced theories of community history legacy effects on microbial community assembly in the context of phenanthrene degradation. Furthermore, it demonstrated that community assembly processes and resulting ecosystem functions at these sites depended on the chronicity of phenanthrene environmental perturbations. Indeed, only high levels of phenanthrene perturbation allowed pre-adaptation of communities to acute perturbation, and short timescales following perturbation may be insufficient to achieve community stability. This information significantly advances our understanding of the microbial communities responsible for degradation of pollutants and is therefore important for both informed responses to remediation following oil spills and assessment of environmental impacts.

## Supporting Information Available

Additional experimental details and results, including statistical analyses (PDF)
