# Supplementary material for: Chronic Environmental Perturbation Influences Microbial Community Assembly Patterns
Source: Environ Sci Technol. 2022 Feb 1;56(4):2300–11. doi: 10.1021/acs.est.1c05106 (PMC9007448; doi:10.1021/acs.est.1c05106)
Supplement: Supplementary file 1 — es1c05106_si_001.pdf [file es1c05106_si_001.pdf]

Chronic environmental perturbation influences microbial community assembly patterns

Lloyd D Potts[1,2*], Alex Douglas[1], Luis J Perez Calderon[1,2], James A Anderson[2], Ursula Witte[1], James I Prosser[1] and Cécile Gubry-Rangin[1]

[1]School of Biological Sciences, University of Aberdeen, Aberdeen AB24 3FX, United Kingdom

[2]Materials and Chemical Engineering, School of Engineering, University of Aberdeen, Aberdeen AB24 3FX, United Kingdom

Number of pages: 27

Number of figures: 6

Number of tables: 2

Supplementary Text

Site nomenclature:

Individual samples were coded as followed: **Site ID_treatment_time point_replicate**, with:

Site ID indicating location of sediment as listed in Supplementary Table 1;

Treatment indicating control or phenanthrene amendments: C/P;

Time points: day 0 = 0, day 28 = 1, day 56 = 2;

Replicates: numbered 1 to 7.

Example: Tyne sediment, phenanthrene-treated, day 28, replicate 5 = TY_P_1_5

Statistical tests and results:

All tests were run in R using the models presented and results output are presented below:

- **Statistic 1: Effect of treatment (control or phenanthrene) on Shannon diversity (H') in pristine and polluted sites over time (days 0 and 28):**

In this analysis, we used a linear mixed effects model to model community alpha-diversity and include Treatment, Day and an indicator variable HC to denote polluted and pristine sites as fixed effects. The HC indicator variable was constructed by labelling the sites as either pristine or polluted as denoted in figure 2 of the manuscript. We included a three-way interaction between these variables (and all associated two-way interactions) in this model to determine whether alpha diversity changes over time (0 and 28 days), whether this difference is dependent on the treatment (control and phenanthrene) and whether these differences are different between polluted and pristine sites. We included a Site random effect to account for variances associated with site in the model. As data at day 56 was not collected for 'polluted' sites we restricted this initial analysis to Day 0 and Day 28 data. A follow up analysis where we analysed pristine sites across days 0, 28 and 56 is presented in statistic 2.

We first excluded day 56 data from the original dataframe:

```
env.028 <- env[env$Fday != "56",]
env.028$Fday <- factor(env.028$Fday)
```

We also removed one replicate at the North Sea pristine site for Day 0, as this extremely low Shannon estimate was unlikely to be an accurate reflection of diversity at this site and probably resulted from a PCR or sequencing error.

```
env <- env[-which(env$Sites_HC == "North_Sea" & env$Fday == "0" &
               env$Shannon < 3),]
```

Then we explored several linear models (and checked their assumptions) and the models tested were the following ones:

```
env.lm1 <- lm(Shannon ~ Treatment * Fday * HC, data = env.028)
```

```
env.lme1 <- lme(Shannon ~ Treatment * Fday * HC, random = ~ 1| Sites_HC, data = env.028)
```

```
env.lme2 <- lme(Shannon ~ Treatment * Fday * HC, random = ~ Fday|Sites_HC,data = env.028)
```

```
env.lme3 <- lme(Shannon ~ Treatment * Fday * HC, random = ~ Fday| Sites_HC, data = env.028,
weights = varIdent(form = ~ 1|Fday))
```

We selected the model env.lme3 by performing a likelihood ratio test (LRT) and by using AIC.

```
anova(env.lme1, env.lme2, env.lme3)

##          Model df      AIC      BIC     logLik   Test  L.Ratio p-value
## env.lme1     1 10 531.2577 567.0927 -255.62886
## env.lme2     2 12 409.6022 452.6042 -192.80111 1 vs 2 125.6555  <.0001
## env.lme3     3 13 190.1595 236.7449  -82.07974 2 vs 3 221.4427  <.0001
```

We validated the best model (env.lme3) after checking the assumptions:

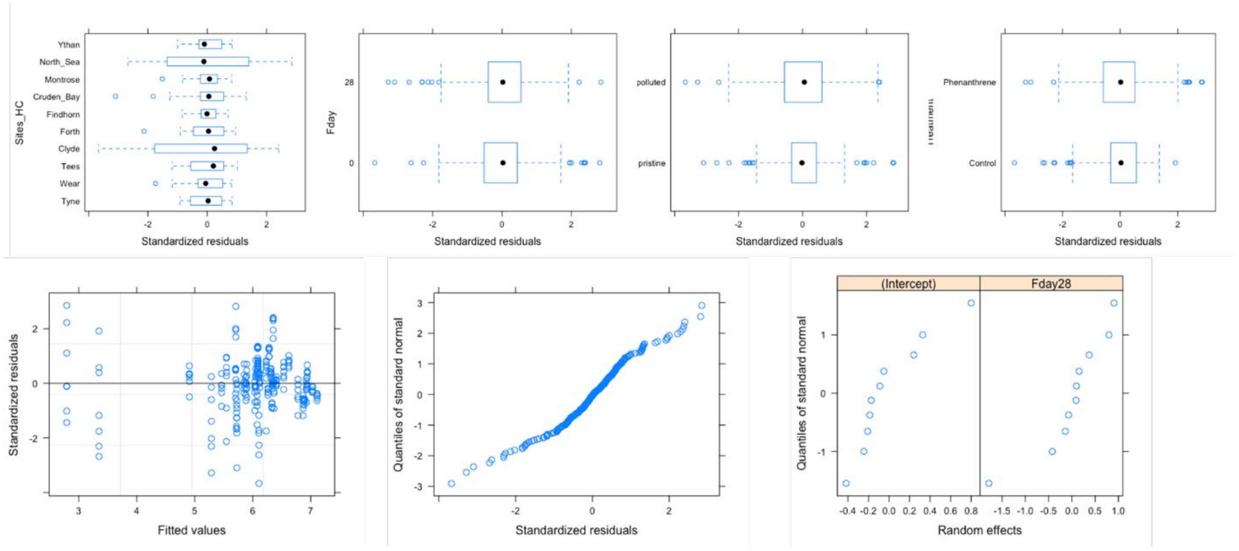

Then, we generated an ANOVA table to interpret the model:

```
round(anova(env.lme3))
```

	numDF	denDF	F-value	p-value
(Intercept)	1	258	2883.2900	0.0000
Treatment	1	258	13.8286	0.0002
Fday	1	258	3.6448	0.0574
HC	1	8	0.7006	0.4269
Treatment:Fday	1	258	57.9279	0.0000
Treatment:HC	1	258	30.6115	0.0000
Fday:HC	1	258	1.9531	0.1635
Treatment:Fday:HC	1	258	5.8033	0.0167

So we have a significant three-way interaction between Treatment, Fday and HC with a reported *F* statistic of 5.8033 and an associated *P* value of 0.0167. We concluded that **mean Shannon *H'* does appear to differ between treatments and this difference is different over time and whether the samples came from a polluted or pristine site.**

We then used the emmeans() function from the emmeans package to analyse the results of the model and used the emm object with the pairs() function to produce a table of relevant comparisons. The *P* values of these comparisons have been adjusted using Tukey's method to control for type I error rate.

```
emm <- emmeans(env.lme3, specs = ~ Treatment * Fday * HC )

pairs(emm, simple = "Fday", reverse = TRUE)
```

contrast	Treatment	HC	estimate	SE	df	t.ratio	p.value
28 - 0	Control	pristine	-0.5874	0.3770	258	-1.5582	0.1204
28 - 0	Phenanthrene	pristine	-1.1298	0.3762	258	-3.0034	0.0029
28 - 0	Control	polluted	0.3988	0.3766	258	1.0589	0.2906
28 - 0	Phenanthrene	polluted	-0.6461	0.3762	258	-1.7174	0.0871

Therefore, **there is no difference in mean Shannon *H'* between control groups at day 0 and day 28 in either pristine or polluted sites** (*P* values = 0.1204 and 0.2904 respectively). **In the pristine sites there is a significant reduction in mean Shannon *H'* in phenanthrene treated groups between day 0 and day 28** (difference = -1.129, *P* value = 0.003). In comparison, **the reduction in Shannon *H'* in phenanthrene treated groups between day 0 and day 28 in polluted sites was non-significant** (difference = -0.646, *P* value = 0.0871).

```
pairs(emm, simple = "HC", reverse = TRUE)
```

contrast	Treatment	Fday	estimate	SE	df	t.ratio	p.value
polluted - pristine	Control	0	0.1537	0.2439	8	0.6304	0.5460
polluted - pristine	Phenanthrene	0	0.4140	0.2440	8	1.6969	0.1281
polluted - pristine	Control	28	1.1399	0.6354	8	1.7940	0.1106
polluted - pristine	Phenanthrene	28	0.8977	0.6346	8	1.4145	0.1949

```
pairs(emm, simple = "Treatment", reverse = TRUE)
```

contrast	Fday	HC	estimate	SE	df	t.ratio	p.value
Phenanthrene - Control	0	pristine	-0.0137	0.0310	258	-0.4424	0.6586
Phenanthrene - Control	28	pristine	-0.5561	0.1447	258	-3.8419	0.0002
Phenanthrene - Control	0	polluted	0.2465	0.0312	258	7.8918	0.0000
Phenanthrene - Control	28	polluted	-0.7983	0.1436	258	-5.5591	0.0000

- **<u>Statistic 2: Effect of treatment (control or phenanthrene) on Shannon diversity (H') in pristine sites over time (days 0, 28 and 56):</u>**

As for statistic 1, we first removed one replicate at the North Sea pristine site for Day 0, as this extremely low Shannon estimate was unlikely to be an accurate reflection of diversity at this site and probably resulted from a PCR or sequencing error.

```
env <- env[-which(env$Sites_HC == "North_Sea" & env$Fday == "0" &
                  env$Shannon < 3),]
```

We then excluded all polluted sites from the original dataframe:

```
env.prist <- env[env$HC == "pristine",]
```

Then we explored several linear models (and checked their assumptions) and the models tested were the following ones:

```
env.prist.lm1 <- lm(Shannon ~ Treatment * Fday, data = env.prist)

env.prist.lme1 <- lme(Shannon ~ Treatment * Fday, random = ~ 1| Sites_HC, data = env.prist)

env.prist.lme2 <- lme(Shannon ~ Treatment * Fday, random = ~ Fday| Sites_HC, data = env.prist)

env.prist.lme3 <- lme(Shannon ~ Treatment * Fday, random = ~ Fday| Sites_HC, data = env.prist,
weights = varIdent(form = ~ 1|Fday))
```

We selected the model env.lme3 by performing a likelihood ratio test (LRT) and by using AIC.

```
anova(env.prist.lme1, env.prist.lme2, env.prist.lme3)

##                Model df      AIC      BIC     logLik   Test  L.Ratio p-value
## env.prist.lme1     1  8 449.8714 476.2979 -216.93572
## env.prist.lme2     2 13 358.9066 401.8496 -166.45330 1 vs 2 100.9648  <.0001
## env.prist.lme3     3 15 220.5757 270.1253  -95.28787 2 vs 3 142.3309  <.0001
```

We validated the best model (env.lme3) after checking the assumptions:

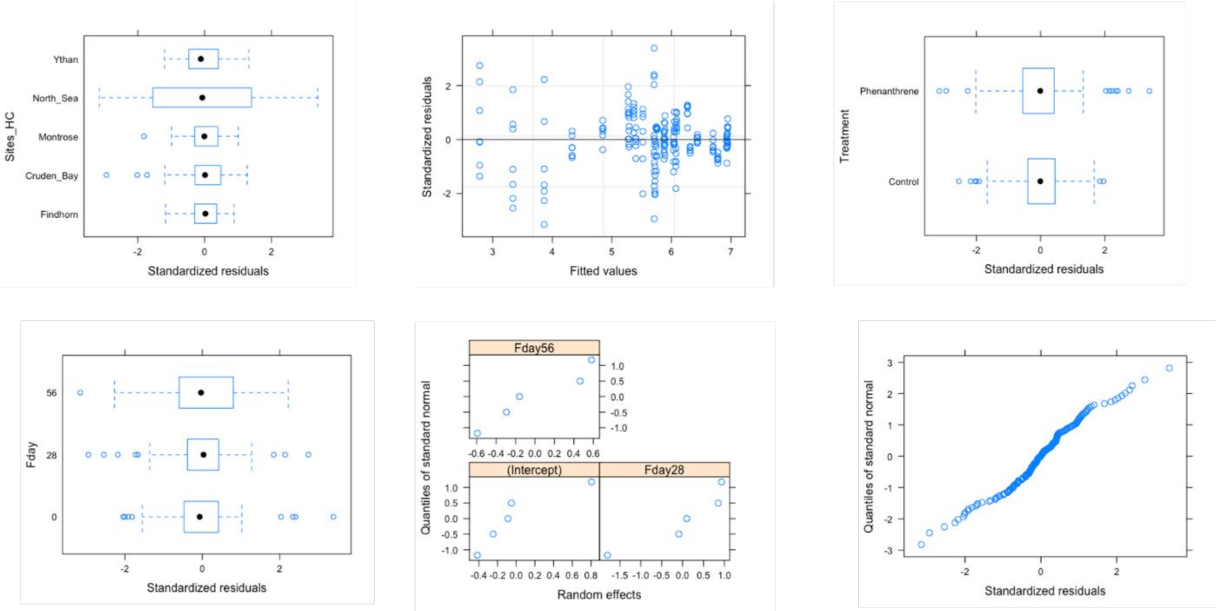

Then, we generated an ANOVA table to interpret the model:

```
round(anova(env.lme3))
```

	numDF	denDF	F-value	p-value
(Intercept)	1	197	924.3565	0.0000
Treatment	1	197	10.0528	0.0018
Fday	2	197	2.6159	0.0756
Treatment:Fday	2	197	60.0940	0.0000

So we have a highly significant two-way interaction between Treatment and Fday with a reported F statistic of 60.094 and an associated P value of P < 0.0001. We concluded that **mean Shannon H' does appear to differ over time and this difference is dependent on the treatment.**

We then used the emmeans() function from the emmeans package to analyse the results of the model and used the emm object with the pairs() function to produce a table of relevant comparisons. The *P* values of these comparisons have been adjusted using Tukey's method to control for type I error rate.

```
emm.prist <- emmeans(env.prist.lme3, specs = ~ Treatment * Fday)

pairs(emm.prist, simple = "Fday", reverse = TRUE)
```

contrast	Treatment	estimate	SE	df	t.ratio	p.value
28 - 0	Control	-0.5876	0.5100	197	-1.1522	0.4833
56 - 0	Control	0.1463	0.2516	197	0.5813	0.8303
56 - 28	Control	0.7339	0.3508	197	2.0922	0.0940
28 - 0	Phenanthrene	-1.1299	0.5093	197	-2.2183	0.0706
56 - 0	Phenanthrene	-1.2508	0.2516	197	-4.9706	0.0000
56 - 28	Phenanthrene	-0.1210	0.3498	197	-0.3458	0.9362

Therefore, **there is no difference in mean Shannon *H'* over time for the control group**. For the phenanthrene-treated group **there is a significant reduction in mean Shannon *H'* between day 0 and day 56** (difference = -1.2508, *P* value = 0.0000).

- **Statistic 3: Effect of treatment (control or phenanthrene) on evenness (estimated by Pielou's J index) in pristine and polluted sites over time (days 0 and 28):**

We applied the same strategy than for statistic 1. We removed one replicate at the North Sea pristine site for Day 0 for consistency between analyses. we explored several linear models (and checked their assumptions) and the models tested were the following ones:

```
env.lm1 <- lm(Pielou ~ Treatment * Fday * HC, data = env.028)

env.lme1 <- lme(Pielou ~ Treatment * Fday * HC, random = ~ 1| Sites_HC, data = env.028)

env.lme2 <- lme(Pielou ~ Treatment * Fday * HC, random = ~ Fday|Sites_HC,data = env.028)

env.lme3 <- lme(Pielou ~ Treatment * Fday * HC, random = ~ Fday| Sites_HC, data = env.028,
weights = varIdent(form = ~ 1|Fday))
```

We selected the model env.lme3 by performing a likelihood ratio test (LRT) and by using AIC.

```
anova(env.lme1, env.lme2, env.lme3)

##         Model df     AIC       BIC   logLik   Test  L.Ratio p-value
```

```
## env.lme1     1 10 -620.9751 -585.1402 320.4876
## env.lme2     2 12 -766.4588 -723.4568 395.2294 1 vs 2 149.4837  <.0001
## env.lme3     3 13 -979.2274 -932.6420 502.6137 2 vs 3 214.7687  <.0001
```

We validated the best model (env.lme3) after checking the assumptions:

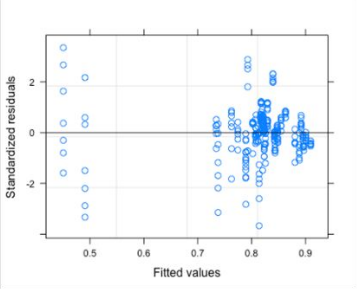

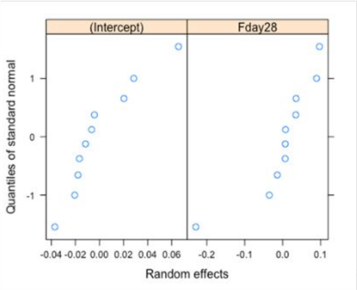

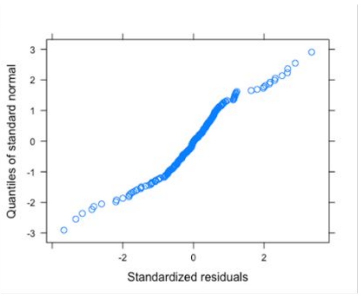

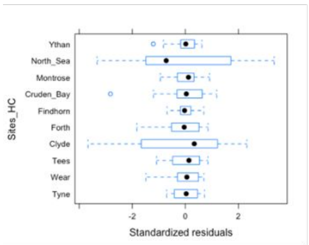

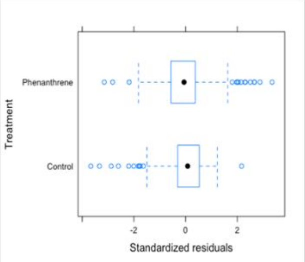

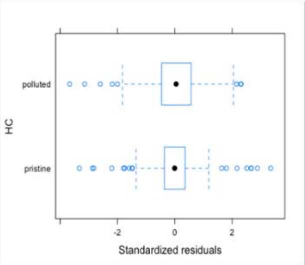

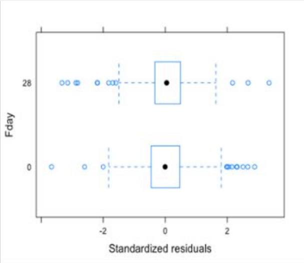

Then, we generated an ANOVA table to interpret the model:

```r
round(anova(env.lme3))
```

	numDF	denDF	F-value	p-value
(Intercept)	1	258	7519.3595	0.0000
Treatment	1	258	20.4762	0.0000
Fday	1	258	2.8541	0.0923
HC	1	8	0.3272	0.5830
Treatment:Fday	1	258	42.2756	0.0000
Treatment:HC	1	258	15.4525	0.0001
Fday:HC	1	258	1.4883	0.2236
Treatment:Fday:HC	1	258	7.2321	0.0076

So we have a significant three-way interaction between Treatment, Fday and HC with a reported *F* statistic of 7.2321 and an associated *P* value of 0.0076. We concluded that **mean Pielou does appear to differ between treatments and this difference is different over time and whether the samples came from a polluted or pristine site.**

We then used the emmeans() function from the emmeans package to analyse the results of the model and used the emm object with the pairs() function to produce a table of relevant comparisons. The *P* values of these comparisons have been adjusted using Tukey's method to control for type I error rate.

```r
Emm.p <- emmeans(env.lme3, specs = ~ Treatment * Fday * HC )

pairs(emm.p, simple = "Fday", reverse = TRUE)
```

contrast	Treatment	HC	estimate	SE	df	t.ratio	p.value
28 - 0	Control	pristine	-0.0693	0.0453	258	-1.5309	0.1270
28 - 0	Phenanthrene	pristine	-0.1131	0.0452	258	-2.5020	0.0130
28 - 0	Control	polluted	0.0390	0.0452	258	0.8626	0.3892
28 - 0	Phenanthrene	polluted	-0.0666	0.0452	258	-1.4737	0.1418

Therefore, **there is no difference in mean Pielou between control groups at day 0 and day 28 in either pristine or polluted sites** (*P* values = 0.1270 and 0.3892 respectively). **In the pristine sites there is a significant reduction in mean Pielou in phenanthrene treated groups between day 0 and day 28** (difference = -2.5020, *P* value = 0.0130). In comparison, **the reduction in Pielou in phenanthrene treated groups between day 0 and day 28 in polluted sites was non-significant** (difference = -1.4737, *P* value = 0.1418).

- **Statistic 4: Effect of treatment (control or phenanthrene) on evenness (estimated by Pielou's J index) in pristine sites over time (days 0, 28 and 56):**

We applied the same strategy than for statistic 2.

We excluded all polluted sites from the original dataframe:

```
env.prist <- env[env$HC == "pristine",]
```

We explored several linear models (and checked their assumptions) and the models tested were the following ones:

```
env.prist.lm1 <- lm(Pielou ~ Treatment * Fday, data = env.prist)

env.prist.lme1 <- lme(Pielou ~ Treatment * Fday, random = ~ 1| Sites_HC, data = env.prist)

env.prist.lme2 <- lme(Pielou ~ Treatment * Fday, random = ~ Fday| Sites_HC, data = env.prist)

env.prist.lme3 <- lme(Pielou ~ Treatment * Fday, random = ~ Fday| Sites_HC, data = env.prist,
weights = varIdent(form = ~ 1|Fday))
```

We selected the model env.lme3 by performing a likelihood ratio test (LRT) and by using AIC.

```
anova(env.prist.lme1, env.prist.lme2, env.prist.lme3)

##                Model df      AIC       BIC   logLik   Test  L.Ratio p-value
## env.prist.lme1     1  8 -407.6732 -381.2467 211.8366

## env.prist.lme2     2 13 -507.7155 -464.7726 266.8578 1 vs 2 110.0424  <.0001

## env.prist.lme3     3 15 -643.3525 -593.8029 336.6762 2 vs 3 139.6369  <.0001
```

We validated the best model (env.lme3) after checking the assumptions:

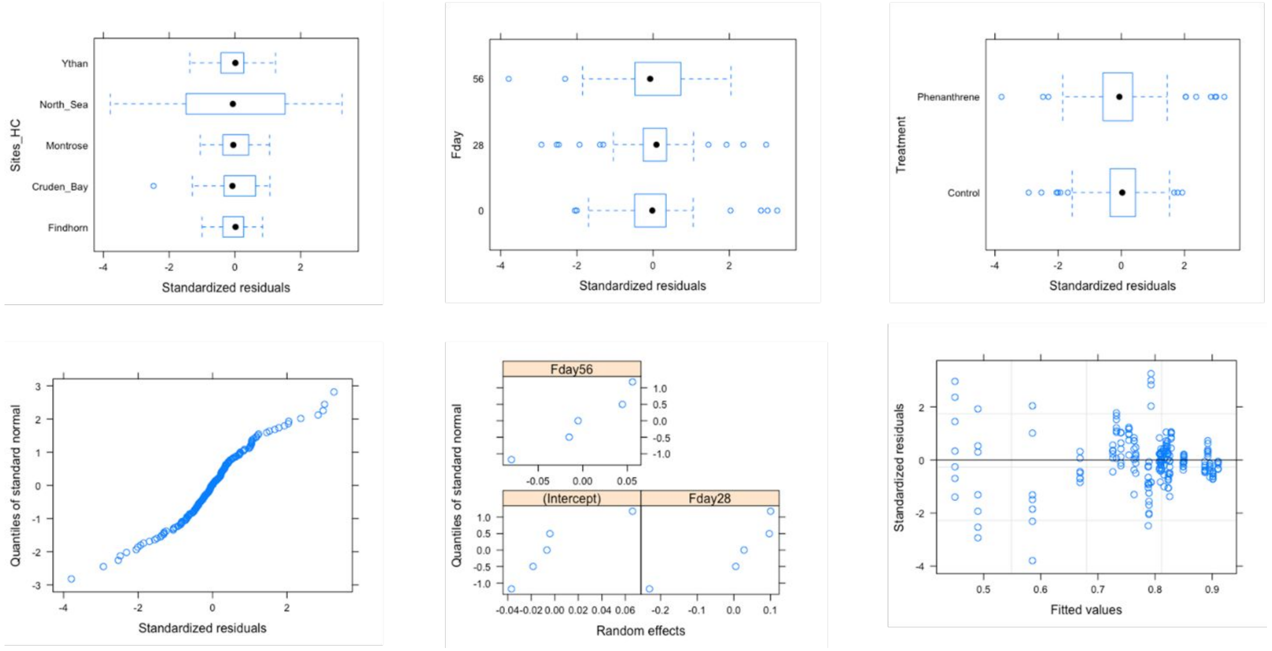

Then, we generated an ANOVA table to interpret the model:

```
round(anova(env.lme3))
```

	numDF	denDF	F-value	p-value
(Intercept)	1	197	2523.5927	0.0000
Treatment	1	197	1.4530	0.2295
Fday	2	197	2.1031	0.1248
Treatment:Fday	2	197	50.7587	0.0000

So we have a highly significant two-way interaction between Treatment and Fday with a reported F statistic of 50.7587and an associated P value of P < 0.0000. We concluded that **mean Pielou does appear to differ over time and this difference is dependent on the treatment.**

We then used the emmeans() function from the emmeans package to analyse the results of the model and used the emm object with the pairs() function to produce a table of relevant comparisons. The *P* values of these comparisons have been adjusted using Tukey’s method to control for type I error rate.

```
emm.prist <- emmeans(env.prist.lme3, specs = ~ Treatment * Fday)

pairs(emm.prist, simple = "Fday", reverse = TRUE)
```

contrast	Treatment	estimate	SE	df	t.ratio	p.value
28 - 0	Control	-0.0694	0.0625	197	-1.1097	0.5092
56 - 0	Control	0.0231	0.0273	197	0.8459	0.6750
56 - 28	Control	0.0924	0.0428	197	2.1603	0.0807
28 - 0	Phenanthrene	-0.1131	0.0624	197	-1.8119	0.1683
56 - 0	Phenanthrene	-0.1274	0.0273	197	-4.6702	0.0000
56 - 28	Phenanthrene	-0.0143	0.0427	197	-0.3361	0.9397

Therefore, **there is no difference in mean Pielou over time for the control group**. For the phenanthrene-treated group **there is a significant reduction in mean Pielou between day 0 and day 56** (difference = -4.6702, *P* value = 0.0000).

- **<u>Statistic 5: Differences between community structure in control and phenanthrene treatments over time</u>**

In this analysis we used a PERMANOVA approach to assess community changes between pristine and polluted sites in control and phenanthrene treatments over time. In order to account for multiple measurements at each site we constrained the permutations within sites.

The original OTU data file had five samples removed (rows 57,79,234,298,318) due to low sequence coverage and then rarefied to 9000 for beta diversity measurements using the rrarefy() function from the vegan package. OTUs with a maximum of zero were subsequently removed from these data. A NMDS was then performed on these OTUs using the metaMDSdist() function and then the betadisper() function was used to reduce the original distances to principal components. The scores were then extracted and added to the data in the comm_data.csv data file as a distance variable.

We first excluded day 56 data from the original dataframe (as only available for the pristine sites):

```
env.028 <- env[env$Fday != "56",]
env.028$Fday <- factor(env.028$Fday)
```

To keep consistency between this analysis and the alpha-diversity analyses, we removed one replicate at the North Sea pristine site for Day 0.

```
env <- env[-which(env$Sites_HC == "North_Sea" & env$Fday == "0" &
                  env$Shannon < 3),]
```

After importing the OTU table into R and removing the low-read samples, we rarefied all samples to 9000 for beta diversity measurements using the rrarefy() function. Once rarefied, the OTUs with a maximum value of 0 were identified and excluded.

```
raremax <- min(rowSums(otu))
```

```
otunorm <- rrarefy(otu, 9000)
ind <- which(colSums(otunorm) == 0)
otunorm <- otunorm[, -ind]
```

Then we created a distance matrix using the NMDS transformation of the otunorm object and subsequently performed the ordination using the betadisper() function and reordered the grouping variables into the two categories (polluted or pristine)

```
dis <- metaMDSdist(otunorm)
```

Fit a NMDS using the Bray-Curtis similarity metric

```
all.mds <- metaMDS(otunorm, trace=FALSE)
```

Then, we assessed the model fit using the stress value, which was fine (stress= 0.1373).

```
stressplot(all.mds)
```

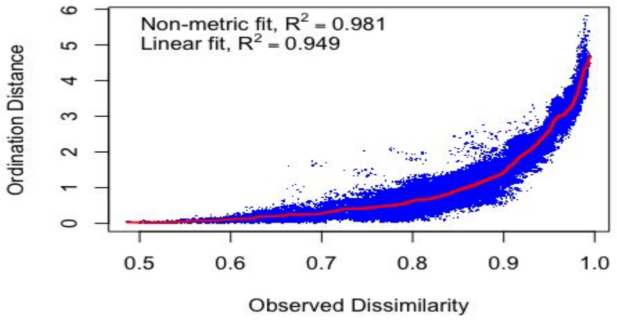

Following this, we performed the PERMANOVA on the distance matrix dis using the adonis() function from the vegan package. We also constrained the permutations by site using the strata = argument.

```
set.seed(001)
sum.ad.strata <- adonis(dis ~ env$Treatment * env$HC * env$Fday, strata = env$Sites_HC)
sum.ad.strata

##
## Call:
## adonis(formula = dis ~ env$Treatment * env$HC * env$Fday, strata = env$Sites_HC)
##
## Blocks:  strata
## Permutation: free
## Number of permutations: 999
##
## Terms added sequentially (first to last)
##
##                                Df SumsOfSqs MeanSqs F.Model      R2 Pr(>F)
## env$Treatment                   1     1.132  1.1319  3.3808 0.00896  0.001 ***
## env$HC                          1     6.711  6.7109 20.0450 0.05314  0.001 ***
## env$Fday                        2     3.472  1.7362  5.1859 0.02750  0.001 ***
## env$Treatment:env$HC            1     0.509  0.5093  1.5211 0.00403  0.001 ***
## env$Treatment:env$Fday          2     1.207  0.6036  1.8028 0.00956  0.001 ***
## env$HC:env$Fday                 1     0.969  0.9689  2.8941 0.00767  0.001 ***
## env$Treatment:env$HC:env$Fday   1     0.466  0.4658  1.3914 0.00369  0.001 ***
## Residuals                     334   111.821  0.3348         0.88545
## Total                         343   126.287                 1.00000
## ---
## Signif. codes:  0 '***' 0.001 '**' 0.01 '*' 0.05 '.' 0.1 ' ' 1
```

We concluded that **community similarity is significantly different between control and phenanthrene treated samples and these differences are dependent on day and whether samples were from pristine and polluted sites** (*P* value = 0.001).

We also assessed one of the assumptions of PERMANOVA, which is that the within group variation (dispersion) is similar between groups and we analysed it using the betadisper() function.

```
anova(betadisper(dis, env$Treatment))

## Analysis of Variance Table
##
## Response: Distances
##            Df  Sum Sq  Mean Sq F value    Pr(>F)
## Groups      1 0.03966 0.039662   12.94 0.0003691 ***
```

```
## Residuals 342 1.04825 0.003065
## ---
## Signif. codes:  0 '***' 0.001 '**' 0.01 '*' 0.05 '.' 0.1 ' ' 1
```

```
anova(betadisper(dis, env$Fday))
```

```
## Analysis of Variance Table
##
## Response: Distances
##            Df  Sum Sq  Mean Sq F value    Pr(>F)
## Groups      2 0.12027 0.060133  17.396 6.399e-08 ***
## Residuals 341 1.17874 0.003457
## ---
## Signif. codes:  0 '***' 0.001 '**' 0.01 '*' 0.05 '.' 0.1 ' ' 1
```

```
anova(betadisper(dis, env$Sites_HC))
```

```
## Analysis of Variance Table
##
## Response: Distances
##            Df  Sum Sq   Mean Sq F value    Pr(>F)
## Groups      9 0.28193 0.0313251  9.5748 5.184e-13 ***
## Residuals 334 1.09272 0.0032716
## ---
## Signif. codes:  0 '***' 0.001 '**' 0.01 '*' 0.05 '.' 0.1 ' ' 1
```

The dispersions are significantly different between control and phenanthrene treatments. There are also differences between day and whether samples from polluted or pristine sites. While the assumption of similar within group dispersions is not met, it has been reported that adonis() is not highly sensitive to dispersion effects (see ?adonis for more details).

- **<u>Statistic 6: Effect of treatment (control or phenanthrene) on community dispersion in pristine and polluted sites over time (days 0 and 28):</u>**

We used a similar approach to statistic 1. In this analysis, we used a linear mixed effects model to model community dispersion and include Treatment, Day and an indicator variable HC to denote polluted and pristine sites as fixed effects. The HC indicator variable was constructed by labelling the sites as either pristine or polluted as denoted in figure 2 of the manuscript. We included a three-way interaction between these variables (and all associated two-way interactions) in this model to determine whether dispersion changes over time (0 and 28 days), whether this difference is dependent on the treatment (control and phenanthrene) and whether these differences are different between polluted and pristine sites. We included a Site random effect to account for variances associated with site in the model. As data at day 56 was not collected for 'polluted' sites we restricted this initial analysis to Day 0 and Day 28 data. A follow up analysis where we analysed pristine sites across days 0, 28 and 56 is presented in statistic 7.

To keep consistency between this analysis and the alpha-diversity analyses, we removed one replicate at the North Sea pristine site for Day 0. We also excluded day 56 data from the original dataframe (as only available for the pristine sites)

We used the distance matrix using the NMDS transformation of the otunorm object estimated in the previous approach (statistic 5). We subsequently performed the ordination using the betadisper() function and reordered the grouping variables into the two categories (polluted or pristine)

```
dis <- metaMDSdist(otunorm)

mod <- betadisper(dis, env$Site, type = c("median","centroid"))
```

Then we explored several linear models (and checked their assumptions) and the models tested were the following ones:

```
env.lm1 <- lm(Distances ~ Treatment * Fday * HC, data = env.028)

env.lme1 <- lme(Distances ~ Treatment * Fday * HC, random = ~ 1| Sites_HC, data = env.028)

env.lme2 <- lme(Distances ~ Treatment * Fday * HC, random = ~ Fday|Sites_HC, data = env.028)

env.lme3 <- lme(Distances ~ Treatment * Fday * HC, random = ~ Fday| Sites_HC, data = env.028,
weights = varIdent(form = ~ 1|Fday))
```

We selected the model env.lme3 by performing a likelihood ratio test (LRT) and by using AIC.

```
anova(env.lme1, env.lme2, env.lme3)

##          Model df       AIC       BIC   logLik   Test  L.Ratio p-value
## env.lme1     1 10 -832.7313 -796.8964 426.3657
## env.lme2     2 12 -922.8349 -879.8329 473.4174 1 vs 2 94.10356  <.0001
## env.lme3     3 13 -988.7559 -942.1704 507.3779 2 vs 3 67.92100  <.0001
```

We validated the best model (env.lme3) after checking the assumptions:

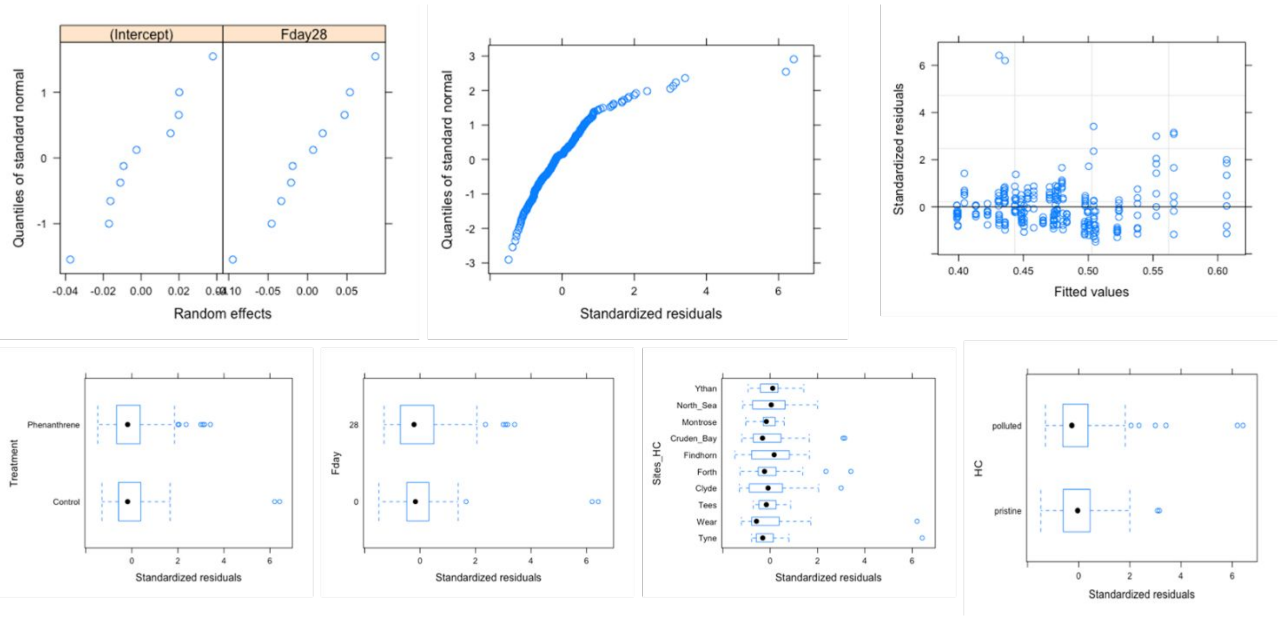

Then, we generated an ANOVA table to interpret the model:

```
round(anova(env.lme3))
```

	numDF	denDF	F-value	p-value
(Intercept)	1	258	5511.1448	0.0000
Treatment	1	258	130.7493	0.0000
Fday	1	258	3.5313	0.0613
HC	1	8	5.1209	0.0535

Treatment:Fday	1	258	2.6172	0.1069
Treatment:HC	1	258	1.7157	0.1914
Fday:HC	1	258	0.4244	0.5153
Treatment:Fday:HC	1	258	10.9251	0.0011

So there is a significant three-way interaction between Treatment, Fday and HC with a reported F statistic of 10.9251 and an associated *P* value of 0.0011. **We concluded that mean dispersion does appear to differ between treatments and this difference is different over time and whether the samples came from a polluted or pristine site.**

We then used the emmeans() function from the emmeans package to analyse the results of the model and used the emm object with the pairs() function to produce a table of relevant comparisons. The *P* values of these comparisons have been adjusted using Tukey's method to control for type I error rate.

```
emm <- emmeans(env.lme3, specs = ~ Treatment * Fday * HC )

pairs(emm, simple = "Fday", reverse = TRUE)
```

contrast	Treatment	HC	estimate	SE	df	t.ratio	p.value
28 - 0	Control	pristine	0.0015	0.0276	258	0.0543	0.9568
28 - 0	Phenanthrene	pristine	0.0443	0.0275	258	1.6094	0.1087
28 - 0	Control	polluted	0.0553	0.0275	258	2.0089	0.0456
28 - 0	Phenanthrene	polluted	0.0408	0.0275	258	1.4817	0.1396

Therefore, there is **no difference in mean dispersion between control groups at day 0 and day 28 in the pristine sites** (P value = 0.7037) and **the mean dispersion is different in the polluted sites between day 0 and day 28** (P value = 0.0456). **In both the pristine and polluted sites, there is no significant difference in mean dispersion in Phenanthrene treated groups between day 0 and day 28** (P value = 0.9568 and 0.1396 respectively).

- **<u>Statistic 7: Effect of treatment (control or phenanthrene) on community dispersion in pristine sites over time (days 0, 28 and 56):</u>**

To keep consistency between this analysis and the alpha-diversity analyses, we removed one replicate at the North Sea pristine site for Day 0. We also excluded all polluted sites from the original dataframe.

We used the dispersion index (mod) estimated in the previous approach (statistic 4).

Then we explored several linear models (and checked their assumptions) and the models tested were the following ones:

```
env.prist.lm1 <- lm(Distances ~ Treatment * Fday, data = env.prist)

env.prist.lme1 <- lme(Distances ~ Treatment * Fday, random = ~ 1| Sites_HC, data = env.prist)

env.prist.lme2 <- lme(Distances ~ Treatment * Fday, random = ~ Fday| Sites_HC, data = env.prist)

env.prist.lme3 <- lme(Distances ~ Treatment * Fday, random = ~ Fday| Sites_HC, data = env.prist,
weights = varIdent(form = ~ 1|Fday), control = lmeControl(msMaxIter = 1e4))
```

We selected the model env.lme3 by performing a likelihood ratio test (LRT) and by using AIC.

```
anova(env.prist.lme1, env.prist.lme2, env.prist.lme3)

##                Model df       AIC       BIC  logLik   Test  L.Ratio p-value
## env.prist.lme1     1  8 -620.9577 -594.5313 318.4789
## env.prist.lme2     2 13 -698.7565 -655.8136 362.3783 1 vs 2 87.79880  <.0001
## env.prist.lme3     3 15 -767.2644 -717.7148 398.6322 2 vs 3 72.50787  <.0001
```

We validated the best model (env.lme3) after checking the assumptions:

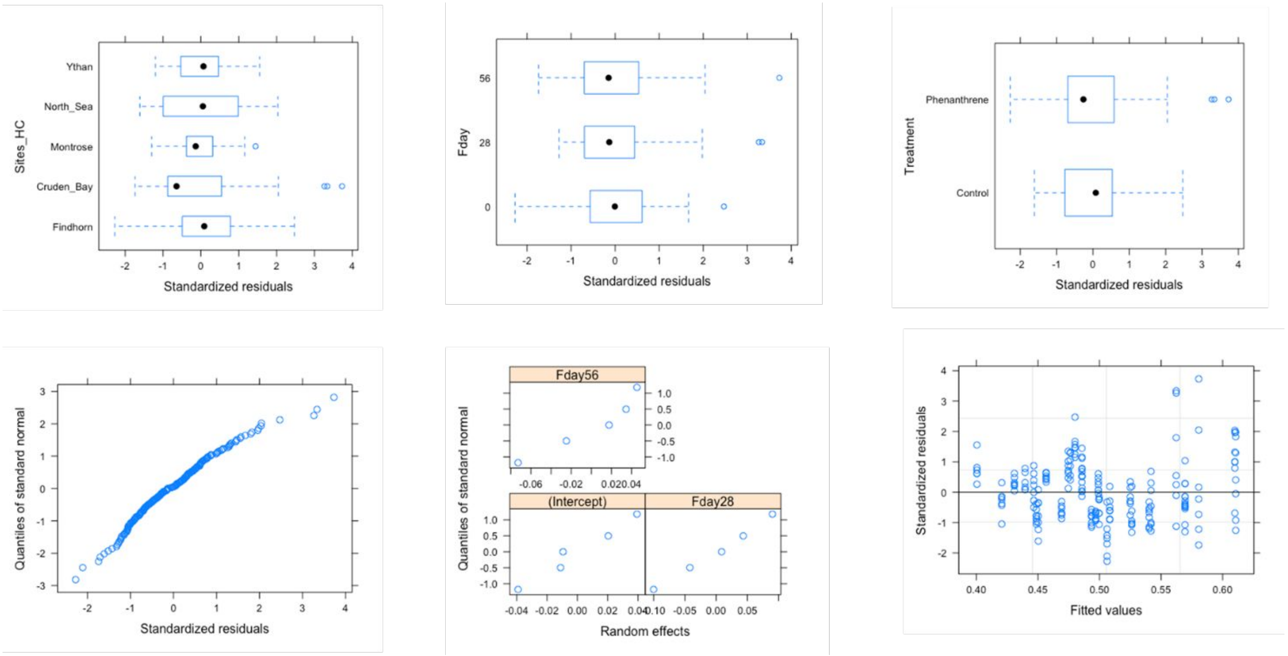

Then, we generated an ANOVA table to interpret the model:

```
anova(env.prist.lme3)
```

	numDF	denDF	F-value	p-value
(Intercept)	1	197	6519.8423	0e+00
Treatment	1	197	133.2963	0e+00
Fday	2	197	9.9514	1e-04
Treatment:Fday	2	197	21.5583	0e+00

So we have a highly significant two-way interaction between Treatment and Fday with a reported F statistic of 21.5583 and an associated *P* value of $P < 0.0001$. We concluded that **mean dispersion does appear to differ over time and this difference is dependent on the treatment.**

We then used the emmeans() function from the emmeans package to analyse the results of the model and used the emm object with the pairs() function to produce a table of relevant comparisons. The *P* values of these comparisons have been adjusted using Tukey's method to control for type I error rate.

```
emm.prist <- emmeans(env.prist.lme3, specs = ~ Treatment * Fday)

pairs(emm.prist, simple = "Fday", reverse = TRUE)
```

contrast	Treatment	estimate	SE	df	t.ratio	p.value
28 - 0	Control	0.0015	0.0344	197	0.0434	0.9990

56 - 0	Control	0.0305	0.0232	197	1.3125	0.3899
56 - 28	Control	0.0290	0.0187	197	1.5532	0.2686
28 - 0	Phenanthrene	0.0443	0.0344	197	1.2875	0.4039
56 - 0	Phenanthrene	0.0882	0.0232	197	3.7972	0.0006
56 - 28	Phenanthrene	0.0439	0.0186	197	2.3662	0.0494

Therefore, **for the control group there is no difference in mean dispersion between each day**. **For the phenanthrene treatment there is a significant difference between days 0 and 56 and days 28 and 56** (*P* values = 6^{-4} and 0.0494 respectively). However, **there is no difference between mean dispersion between days 0 and 28** (*P* value = 0.4039).

- **Statistic 8: phylogenetic signal in the dataset**

From the OTU table (not normalised to 9000 reads per sample), we rarefied each sample to 500 reads:

```
otunorm2 <- rrarefy(otu2, 500)

otunorm2[1:5,1:5]

w = which(colSums(otunorm2) == 0)

otunorm3 = otunorm2[,-w]

write.csv(otunorm3, "otunorm3.csv", quote = F)
```

We then selected the 1000 most abundant OTUs in excel and the result is otunorm4.csv

We subsequently built a tree on a Biolinux server using the following commands:

```
mafft --auto Lloyd_rarefied.fasta > Lloyd_rarefied_mafft.phy

trimal -in Lloyd_rarefied_mafft.phy -out Lloyd_rarefied_trimed.fasta -gt 0.5

iqtree -s Lloyd_mafft_trimed.fasta -alrt 1000 -bb 1000
```

The tree was then converted in nexus format in figtree and reimported in R

```
phylo <- read.nexus('Lloyd.nex')
```

The phylogenetic tree was then matched to the meanTPH concentration, which was estimated as the ponderated mean of TPH initial (sediment) concentration in function of the abundance of reads within each site.

```
tph_mean <- read.csv('OTU_TPHpref.csv')

taxa<- data[,1]

tph<- data[,2]

dim(tph_mean)
```

```
tph <- tph_mean$taxa
```

The phylogenetic signal was then assessed using the following commands:

```
match.phylo.data(phylo,taxa)

phylosignal(tph_mean$tph, phylo, reps = 999, checkdata=TRUE)
```

The mantel correlogram was plotted used the function mantel.correlog from the vegan package:

```
mantel.correlog(D.eco = n, D.geo = x, n.class = 50, cutoff = FALSE, nperm = 999, mult =
"bonferroni")
```

- **Statistic 9: Phenanthrene degradation over time in pristine versus polluted sediments**

Due to the destructive sampling approach required for the phenanthrene quantification, phenanthrene degradation was only estimated for polluted sites at day 28 and for pristine sites at day 56. As such, the pristine sites received a double amount of phenanthrene compared to the polluted sites in order to account for the additional time. To take into account the differences in the initial concentration, we calculated the percentage degradation ((start conc - end conc) / start conc) instead of using the final concentration.

Then we explored several linear models (and checked their assumptions) and the models tested were the following ones:

```
end.lme1 <- lme(Percent ~ Polluted, random = ~ 1|Fsite, data = end)

end.lme2 <- lme(Percent ~ Polluted, random = ~ 1|Fsite, weights = varIdent(form = ~1|Fsite), data
= end)
```

We selected the model end.lme2 by performing a likelihood ratio test (LRT) and by using AIC.

```
anova(end.lme1, end.lme2)

##          Model df      AIC      BIC    logLik   Test  L.Ratio p-value
## end.lme1     1  4 386.9413 395.8193 -189.4706
## end.lme2     2 13 367.8783 396.7319 -170.9391 1 vs 2 37.06301  <.0001
```

We validated the best model (end.lme2) after checking the assumptions:

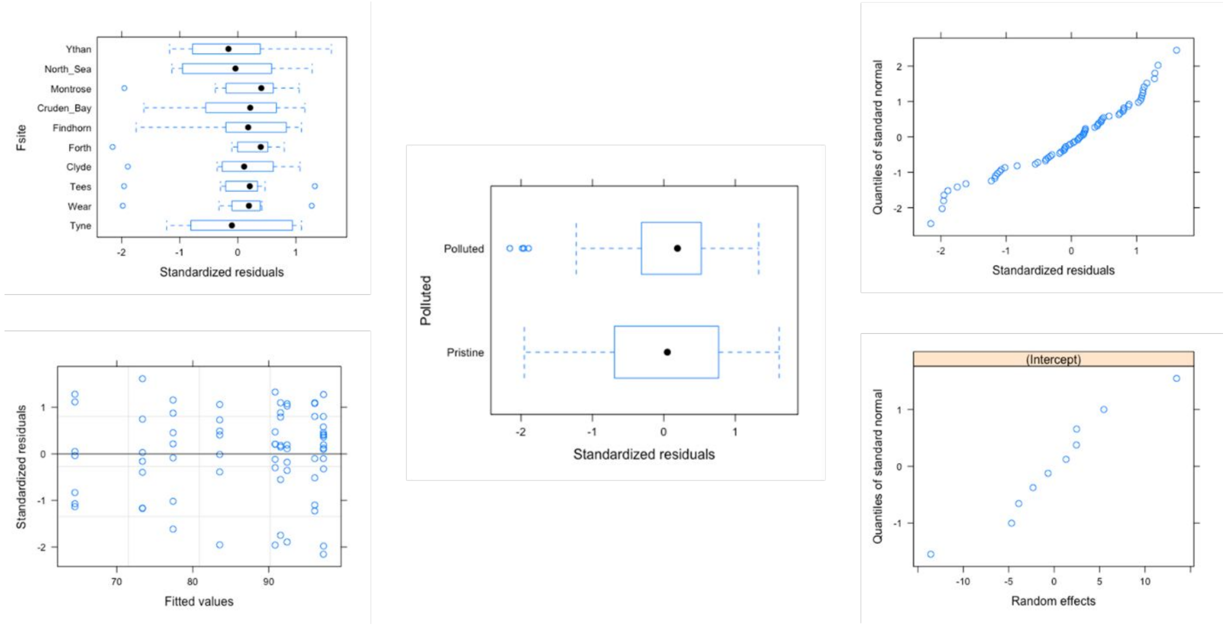

Then, we generated an ANOVA table to interpret the model:

```
anova(end.lme2)
```

	numDF	denDF	F-value	p-value
(Intercept)	1	60	1269.2633	0.000
Polluted	1	8	11.7563	0.009

There is a significant difference in mean phenanthrene degradation between pristine and polluted sites (*F* = 11.76, *P* value = 0.009).

Finally, we inspected the summary output:

```
summary(end.lme2)

## Linear mixed-effects model fit by REML
##   Data: end
##        AIC      BIC    logLik
##   367.8783 396.7319 -170.9391
##
## Random effects:
##  Formula: ~1 | Fsite
##         (Intercept) Residual
## StdDev:    7.599983 1.644172
##
## Variance function:
##  Structure: Different standard deviations per stratum
##  Formula: ~1 | Fsite
##  Parameter estimates:
##       Tyne       Wear       Tees      Clyde      Forth   Findhorn Cruden_Bay
##  1.0000000  0.8209656  1.4606762  0.4087936  0.9552253  2.0706137  1.3273985
##   Montrose  North_Sea      Ythan
##  2.1024062  1.6392265  3.8126826
## Fixed effects:  Percent ~ Polluted
##                    Value Std.Error DF   t-value p-value
## (Intercept)     78.07337  3.459705 60 22.566481   0.000
```

```
## PollutedPolluted 16.65565  4.857652  8  3.428744   0.009
##  Correlation:
##                 (Intr)
## PollutedPolluted -0.712
##
## Standardized Within-Group Residuals:
##        Min         Q1        Med         Q3        Max
## -2.1556599 -0.3947771  0.1343201  0.7409681  1.6124580
##
## Number of Observations: 70
## Number of Groups: 10
```

From the table of parameter estimates we can see that **on average 78.07% phenanthrene is degraded in the pristine sites**. **In polluted sites, degradation is 16.66% higher compared to pristine sites**.

Supplementary Table 1. Characteristics of the sampled sites.

Site	Site ID	TPH (μg g^{-1})	Site category	Phenanthrene (μg g^{-1})	Latitude	Longitude
Tyne	TY	3.84	'polluted'	0.71	54.961988	-1.544336
Wear	WE	1.10	'polluted'	0.36	54.913411	-1.404507
Tees	TS	0.60	'polluted'	0.14	54.572430	-1.262425
Clyde	CL	0.29	'polluted'	n.d	55.916531	-4.460331
Forth	FO	0.28	'polluted'	n.d	56.016645	-3.618900
Findhorn	FH	0.13	'pristine'	n.d	57.638099	-3.582515
Montrose	MO	n.d	'pristine'	n.d	56.721167	-2.486692
Cruden_Bay	CB	n.d	'pristine'	n.d	57.415162	-1.848125
North_Sea	NS	n.d	'pristine'	n.d	55.915736	-1.446019
Ythan	YT	n.d	'pristine'	n.d	57.331221	-1.995985

TPH = μg total petroleum hydrocarbon g^{-1} sediment

n.d = not detected (<0.1 μg g^{-1})

Supplementary Table 2: Heat map illustrating the standard deviation (as a percentage of the whole community; all sites and time points *n*=7, except for Wear day 0 and Clyde day 0, *n*=6) corresponding to values shown in Fig 5, illustrating the relative abundance (as a percentage of the whole community) of the 20 most abundant taxa at the family level in phenanthrene-treated communities from each site. Scale bar indicates colour range used for percentage abundance.

	Polluted										Pristine														
Site	Tyne		Wear		Tees		Clyde		Forth		Findhorn			Cruden Bay			Montrose			North Sea			Ythan		
Time (days)	0	28	0	28	0	28	0	28	0	28	0	28	56	0	28	56	0	28	56	0	28	56	0	28	56
Nitrosopumilaceae	0.0	0.0	0.0	0.1	0.1	1.0	0.1	0.0	0.0	0.0	0.0	0.0	0.0	0.1	0.1	0.1	0.0	0.0	0.0	9.1	1.9	1.7	0.0	0.0	0.0
Mycobacteriaceae	0.1	0.0	0.1	0.1	0.0	0.0	0.0	0.2	0.0	0.1	0.0	2.8	11.5	0.0	0.1	0.7	0.0	0.2	1.3	0.0	0.0	0.0	0.0	0.0	0.1
Micrococcaceae	0.0	0.0	0.0	0.0	0.0	0.0	0.1	11.4	0.0	6.5	0.0	0.0	0.0	0.2	3.3	2.1	0.0	0.2	1.9	0.0	0.0	0.0	0.0	0.0	0.0
Flavobacteriaceae	0.4	0.7	0.9	1.2	0.3	0.3	0.7	1.6	0.3	0.9	0.3	2.2	1.7	0.8	3.6	1.2	0.7	0.8	0.7	0.2	1.0	1.1	0.8	0.8	1.0
Pirellulaceae	0.1	0.1	0.3	0.9	0.3	0.3	0.7	0.9	0.3	1.4	0.2	0.5	0.7	0.2	1.1	1.0	0.4	0.7	0.6	2.0	0.3	0.5	0.3	0.9	0.6
Rhodobacteraceae	0.1	0.2	0.3	1.6	0.3	0.2	0.7	3.0	0.2	8.0	0.4	0.9	0.5	0.4	3.2	0.6	0.5	0.3	0.9	0.1	18.5	9.7	0.6	0.3	0.2
Sphingomonadaceae	0.0	0.2	0.2	0.1	0.1	0.1	0.3	0.1	0.1	1.1	0.0	2.7	3.9	0.1	0.8	2.1	0.0	3.5	3.3	0.0	14.8	8.9	0.0	0.1	1.4
Desulfobulbaceae	0.3	0.5	0.2	1.7	0.1	0.3	0.2	0.1	0.2	1.3	0.3	0.4	0.2	0.1	0.2	0.2	0.3	0.1	0.1	0.0	0.0	0.2	0.3	0.2	0.2
Alteromonadaceae	0.0	0.0	0.0	1.3	0.0	0.0	0.0	0.0	0.0	2.6	0.0	1.6	0.8	0.0	0.0	0.0	0.0	0.2	0.1	0.0	7.7	13.7	0.0	0.0	0.0
Pseudoalteromonadaceae	0.0	0.1	0.0	0.0	0.0	0.0	0.0	0.0	0.0	0.0	0.0	0.0	0.0	0.0	0.0	0.0	0.0	0.0	0.0	16.8	3.6	2.5	0.0	0.0	0.0
Burkholderiaceae	0.1	0.4	0.1	0.1	0.0	0.1	0.7	4.9	0.1	0.2	0.0	1.5	5.5	0.1	5.1	3.7	0.0	1.8	4.0	0.1	0.0	1.4	0.1	1.7	4.5
Halieaceae	0.2	0.1	0.3	0.4	0.1	0.2	0.9	0.3	0.4	0.7	0.2	0.1	0.1	0.3	0.4	0.3	0.1	0.4	0.3	0.2	0.2	0.1	0.5	0.1	0.1
Porticoccaceae	0.0	0.3	0.0	1.9	0.1	0.1	0.0	0.0	0.0	2.6	0.0	0.0	0.0	0.0	0.3	0.1	0.0	0.0	0.1	0.0	6.6	7.6	0.0	0.0	0.0
Gammaproteobacteria	0.2	0.5	0.5	1.0	0.2	0.5	0.2	0.1	0.3	2.5	0.2	1.2	0.2	0.5	0.5	0.4	0.3	0.5	1.1	1.5	0.8	0.4	0.3	0.1	0.2
Piscirickettsiaceae	0.0	0.5	0.0	3.2	0.0	1.7	0.0	0.0	0.0	5.2	0.0	0.0	0.0	0.0	0.0	0.0	0.0	3.9	6.1	0.0	20.1	24.7	0.0	0.0	0.0
Moraxellaceae	0.0	0.0	0.0	0.0	0.0	0.0	0.8	3.7	0.0	0.0	0.2	0.0	0.0	1.1	4.3	0.9	1.0	0.0	0.0	0.1	0.1	0.0	0.8	6.0	0.0
Pseudomonadaceae	0.0	0.0	0.0	0.9	0.0	0.0	0.2	1.0	0.0	1.5	0.0	1.5	1.0	0.1	4.6	1.8	0.0	0.2	0.1	0.0	0.0	0.1	2.1	0.6	0.2
Arcobacteraceae	0.0	0.0	0.0	0.0	0.0	0.0	0.0	0.0	0.0	0.2	0.1	2.1	5.7	0.0	0.0	0.0	0.1	2.4	0.3	0.0	0.0	0.0	0.1	0.1	0.0
Thiovulaceae	0.2	0.2	0.9	0.5	0.0	0.0	0.0	0.0	0.0	0.6	0.0	0.8	5.2	0.0	0.0	0.0	0.0	0.7	0.1	0.0	0.0	0.0	0.0	0.0	0.0
Anaerolineaceae	0.1	0.4	0.4	0.7	0.1	0.4	0.2	0.4	0.2	0.6	0.1	0.3	0.1	0.3	0.4	0.4	0.1	0.2	0.2	0.4	0.1	0.2	0.0	0.1	0.2

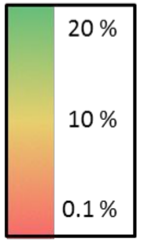

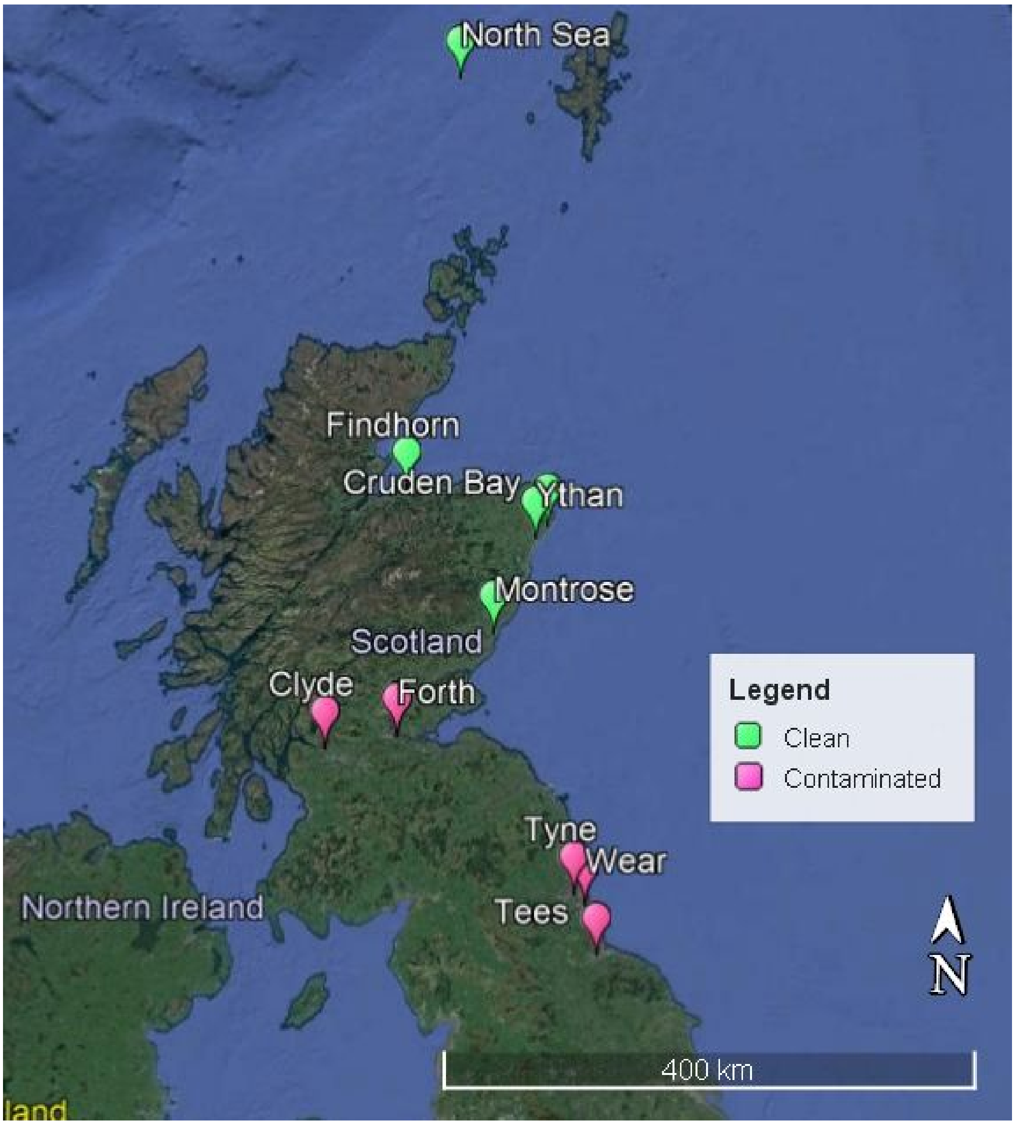

Supplementary Figure 1. Geographical representation of the 10 selected UK sites.

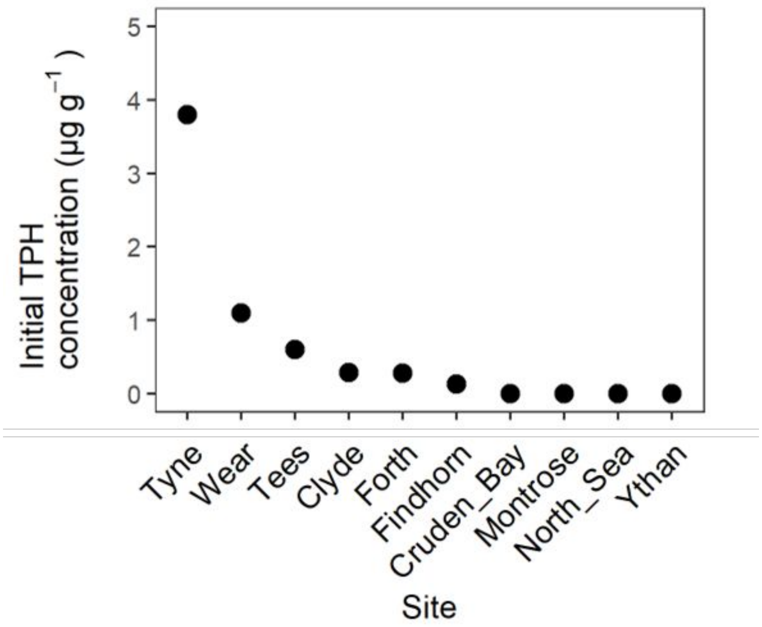

Supplementary Figure 2. Total petroleum hydrocarbon (TPH) concentration in sediments prior to incubation, with sites ordered along the x-axis with decreasing initial TPH concentration.

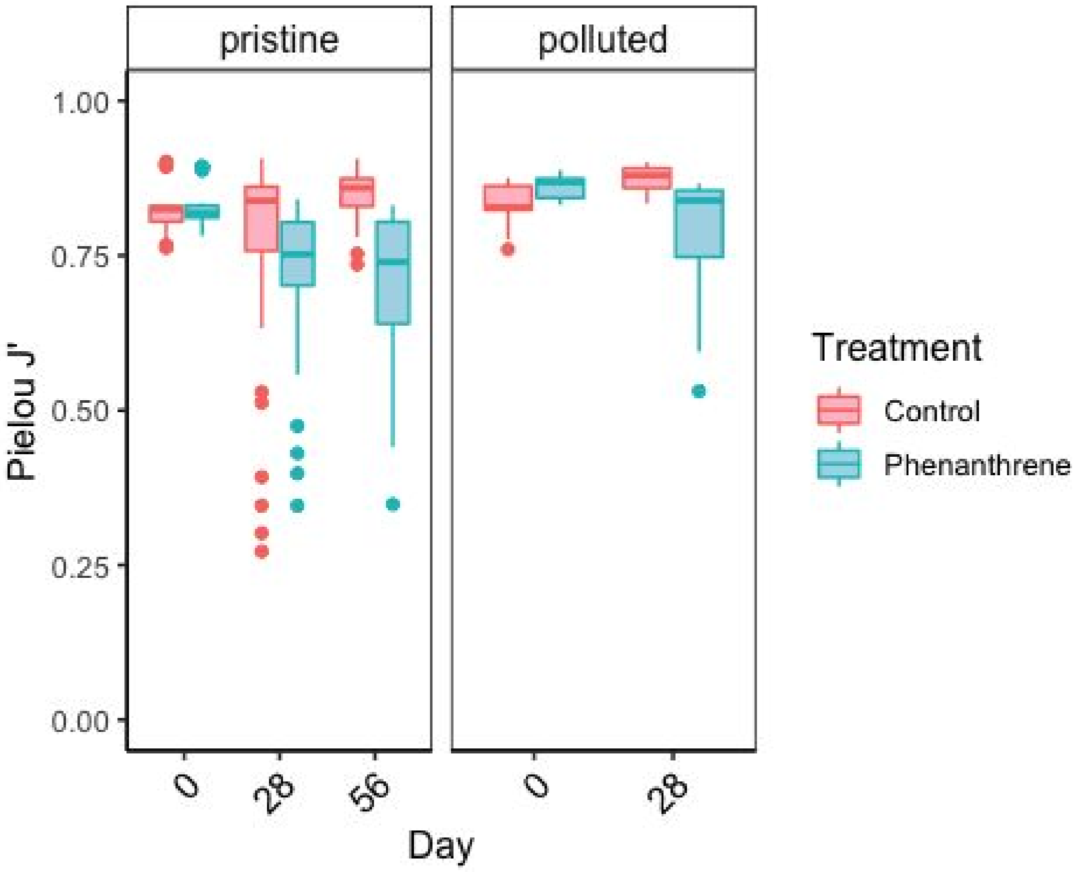

Supplementary Figure 3. Estimated alpha diversity (Shannon index) across all the pristine and polluted sites in control and phenanthrene-treated communities over time; only the pristine communities were incubated for 56 days.

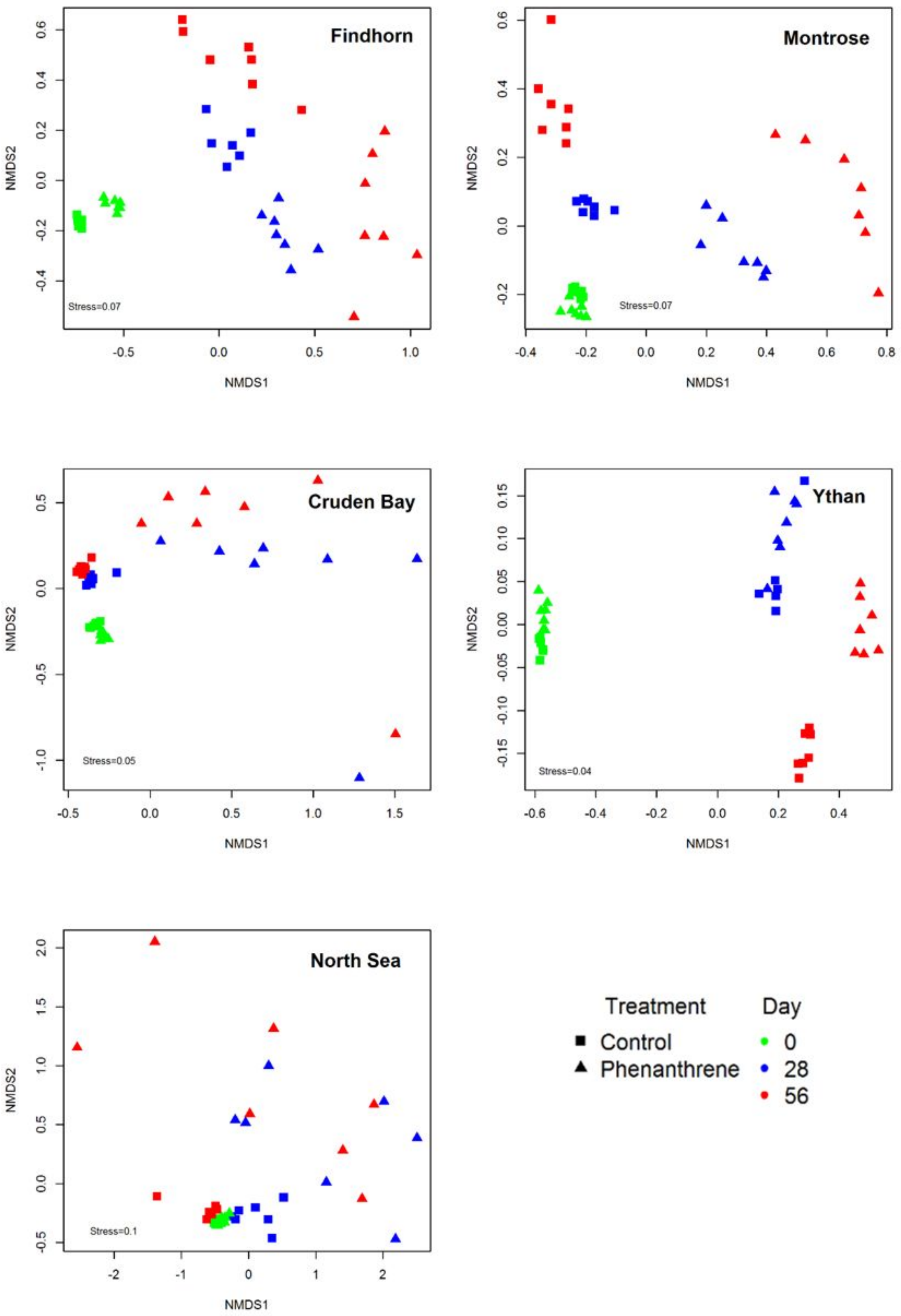

Supplementary Figure 4. Ordination (non-metric multi-dimensional scaling; nMDS) of all treatments and time points for each pristine site, indicating the level of within-site dispersion between replicates over time.

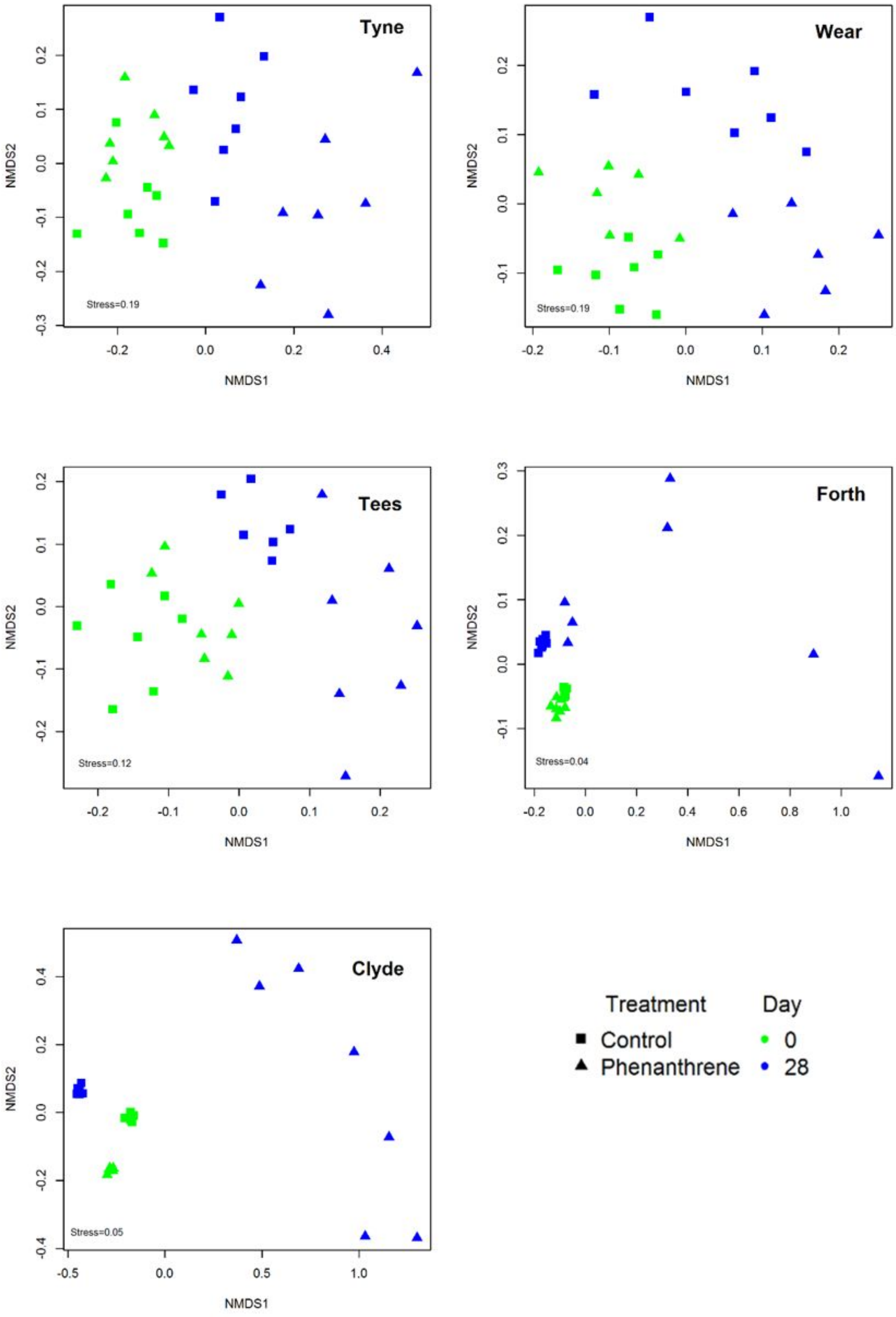

Supplementary Figure 5. Ordination (non-metric multi-dimensional scaling; nMDS) of all treatments and time points for each polluted site, indicating the level of within-site dispersion between replicates over time.

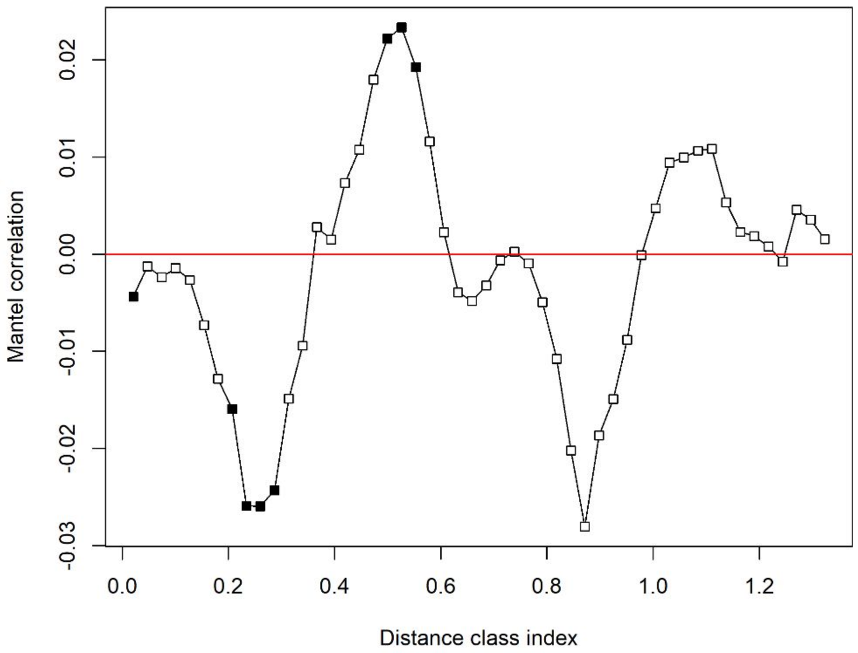

Supplementary Figure 6. Phylogenetic Mantel correlogram relating OTU niche differences (i.e. optimal TPH concentrations for each OTU) to between-OTU phylogenetic distances. Solid and open symbols denote significant and non-significant correlations, respectively.
